# Accessing low-oxidation state taxanes: is taxadiene-4(5)-epoxide on the taxol biosynthetic pathway?†
†Electronic supplementary information (ESI) available: Full experimental procedures and copies of ^1^H and ^13^C NMR spectra. CCDC 1030909. For ESI and crystallographic data in CIF or other electronic format see DOI: 10.1039/c5sc03463a


**DOI:** 10.1039/c5sc03463a

**Published:** 2016-01-26

**Authors:** Naomi A. Barton, Benjamin J. Marsh, William Lewis, Nathalie Narraidoo, Graham B. Seymour, Rupert Fray, Christopher J. Hayes

**Affiliations:** a School of Chemistry , University of Nottingham , University Park , NG7 2RD , Nottingham , UK; b Division of Plant and Crop Sciences , School of Biosciences , University of Nottingham , Sutton Bonnington , LE12 5RD , Loughborough , UK . Email: chris.hayes@nottingham.ac.uk ; Fax: +44 (0)115 951 3564 ; Tel: +44 (0)115 951 3045

## Abstract

We have shown for the first time that taxadiene (**3**) can be epoxidised in a regio- and diastereoselective manner to provide taxadiene-4(5)-epoxide (**12**) as a single diastereoisomer, and that this epoxide can be rearranged to give taxa-4(20),11(12)-dien-5α-ol (**4**). Furthermore, the epoxide **12** rearranges under acidic conditions to give taxa-4(20),11(12)-dien-5α-ol (**4**), the known bridged ether OCT (**5**) and the new oxacyclotaxane (OCT2) **15**. Contrary to previous speculation, taxadiene-4(5)-epoxide (**12**) is susceptible to rearrangement when exposed to an iron^III^ porphyrin, and these observations justify consideration of epoxide **12** as a chemically competent intermediate on the taxol biosynthetic pathway.

## Abstract 2

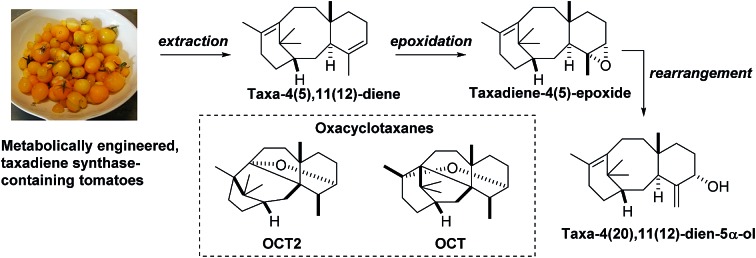
We report the first synthesis of taxadiene-4(5)-epoxide, which rearranges upon acid treatment to produce products of relevance to taxol biosynthesis.

## Introduction

Since its isolation from the pacific yew (*Taxus brevifolia*), and subsequent FDA approval in 1992, taxol and its close derivatives continue to be used as frontline drugs for the treatment of cancer.^1^ Its effectiveness in the clinic, coupled with an intriguing tricyclic structure, has ensured that taxol has endured as a molecule of interest to scientists for nearly 50 years.^2^ In this paper we show that a combination of metabolic engineering and synthetic chemistry can be used to give ready access to low oxidation state taxanes, giving new insight into the early stages of the ‘oxidase-phase’ of the taxol biosynthetic pathway.^3^

**Scheme 1:**
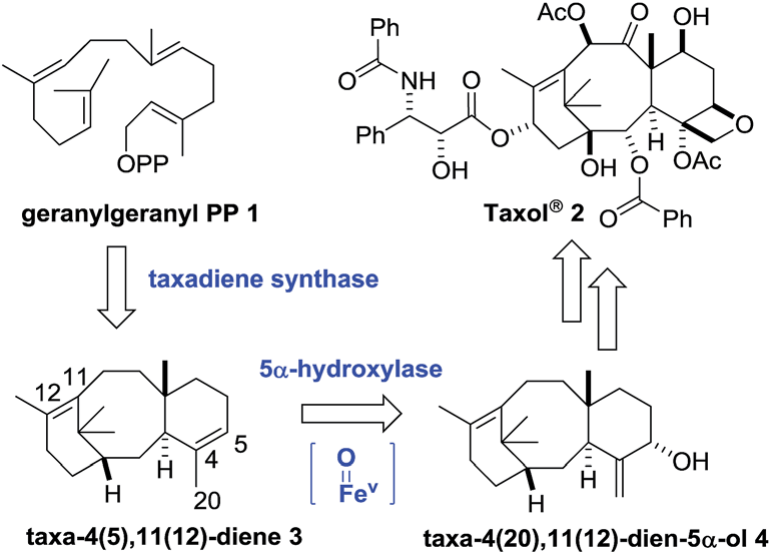
Biosynthesis of Taxol® from geranylgeranyl-pyrophosphate, *via* taxadiene.

The first committed step in the taxol biosynthetic pathway (Scheme 1) is the taxadiene synthase-catalysed cyclisation of geranylgeranyl pyrophosphate **1** to produce taxa-4(5),11(12)-diene (**3**).^4^ The remaining biosynthetic steps involve a series of oxidation, and functional group interconversion processes, the first of which is the taxadiene-5α-hydroxylase-mediated oxidation of **3** into taxa-4(20),11(12)-dien-5α-ol (**4**).^5^

**Scheme 2:**
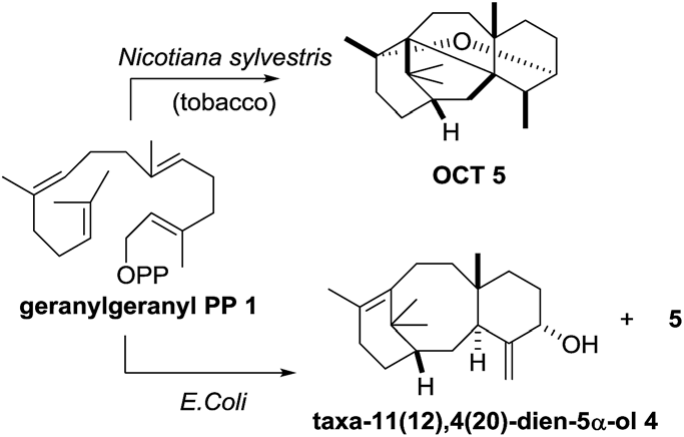
Production of oxidised taxanes in metabolically engineered tobacco and *E. coli* containing both taxadiene synthase and taxadiene hydroxylase.

A number of research groups have reported the over-production of taxa-4(5),11(12)-diene (**3**) in a variety of chassis organisms (yeast,^6^ tobacco,^7^*E. coli*,^8^ tomato^9^), and the incorporation of both taxadiene synthase and its 5α-hydroxylase (tobacco,^7^*E. coli*8*a*) has also been described. In 2008 Rontein showed that overexpression of both taxadiene synthase and taxa-4(5),11(12)-diene 5-hydroxylase (CYP725A4) in tobacco (*Nicotiana sylvestris*) did not produce taxa-4(20),11(12)-dien-5α-ol (**4**) as expected, but instead led to the production of 5(12)-oxa-3(11)-cyclotaxane (OCT) **5** (Scheme 2).^7^

In 2010 Stephanopoulos reported a significant improvement in this area using *E. coli* as the chassis organism.8*a* Under their optimised conditions, taxa-4(20),11(12)-dien-5α-ol (**4**) could be produced, but unfortunately the desired product **4** was obtained as a 1 : 1 mixture with OCT (**5**), thus severely limiting the amount of **4** being produced. These two studies clearly demonstrate that the presence of both taxadiene synthase and taxadiene-5α-hydroxylase in a metabolically engineered chassis organism does not guarantee satisfactory production of taxadien-5-ol **4**, and the catalytic promiscuity and multispecificity of taxadiene-5α-hydroxylase has attracted recent attention.^10^

**Scheme 3:**
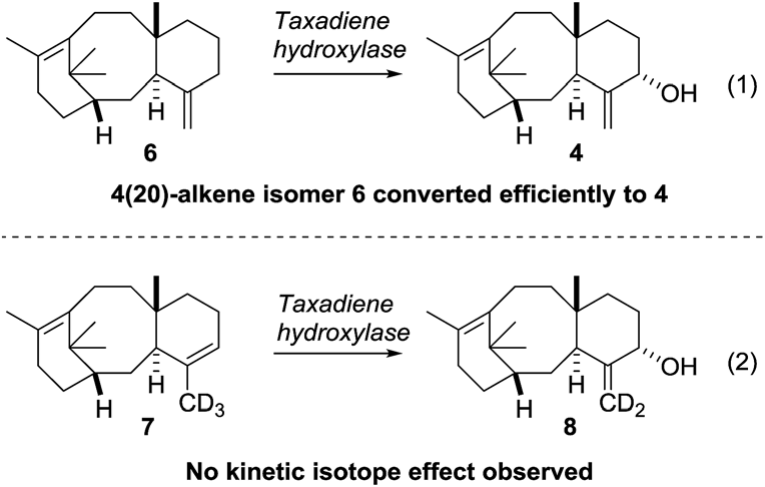
Elucidating the taxadiene hydroxylase mechanism (Williams and Croteau).

**Scheme 4:**
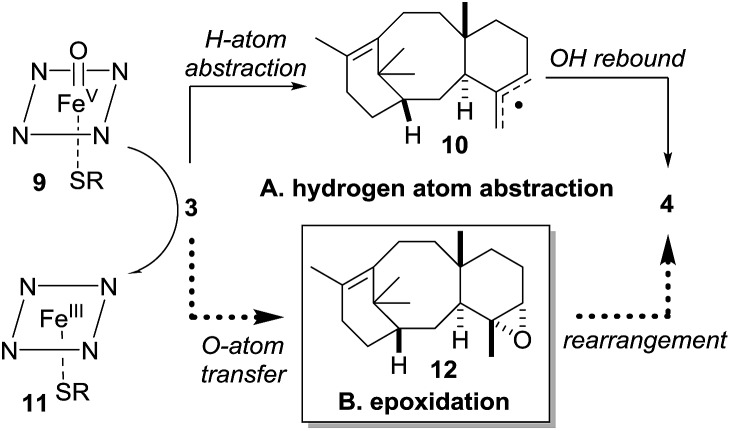
Taxadiene hydroxylase mediated oxidation of taxa-4(5),11(12)-diene (**3**) to taxa-4(20),11(12)-dien-5α-ol (**4**).

Our current understanding of the taxadiene-5α-hydroxylase oxidation mechanism is derived from experiments performed by Williams and Croteau (Scheme 3).^5^ The observation that taxadiene-containing microsomes could convert both the 4(5)-**3** (Scheme 1) and the 4(20)-**6** alkene isomers of taxadiene to taxadien-5-ol **4** with equal efficiency (Scheme 3, eqn (1)), lead Williams and Croteau to suggest an H-atom abstraction/oxygen rebound mechanism, *via* the allylic radical **10**, as being the most likely (path A, Scheme 4).

An alternative pathway involving epoxidation of **3** to give **12**, followed by rearrangement to give **4** (path B, Scheme 4) was also considered, but was eventually discounted by the fact that the 4(20)-alkene isomer **6** is also converted to **4** by taxadiene hydroxylase (*via* a process unlikely to involve **12**).^5^ This conclusion was further supported by the fact that the epoxide **12** has not been observed as an oxidation product of **3** in any studies reported thus far. In order to provide further evidence for the H-atom abstraction/oxygen rebound mechanism (path A, Scheme 4), Williams *et al.* prepared deuterium-labelled [C20–^2^H_3_]-taxadiene (**7**) and subjected this to taxadiene hydroxylase. However, under these conditions, the expected kinetic isotope effect was not observed for the transformation of **7** to **8** (Scheme 3, eqn (2)),^5^ which is at odds with the proposed H-atom abstraction process. Furthermore, Williams *et al.* report that their experiment ‘unexpectedly revealed that the deuterated substrate yielded slightly more taxa-4(20),11(12)-dien-5α-ol than did the unlabeled substrate’,5*b* thus indicating a small inverse isotope effect. This experimental observation actually supports the epoxide/rearrangement route for the conversion of **3** to **4**, as small inverse secondary isotope effects are observed in epoxidation reactions,^11^ but no further experiments have been reported to examine this possibility.

The production of OCT **5**, along with additional oxidation products, in engineered taxadiene synthase/taxadiene hydroxylase-containing organisms^7^,8a lead us to question whether epoxide **12** could be an intermediate in the taxadiene hydroxylase mechanism as we could envisage viable pathways for the production of both **4** and **5** from epoxide **12**. Therefore, we decided to synthesise **12** and study it's chemistry in the context of the early stages of the taxol biosynthetic pathway.

## Results and discussion

### Epoxidation of taxadiene

**Scheme 5:**
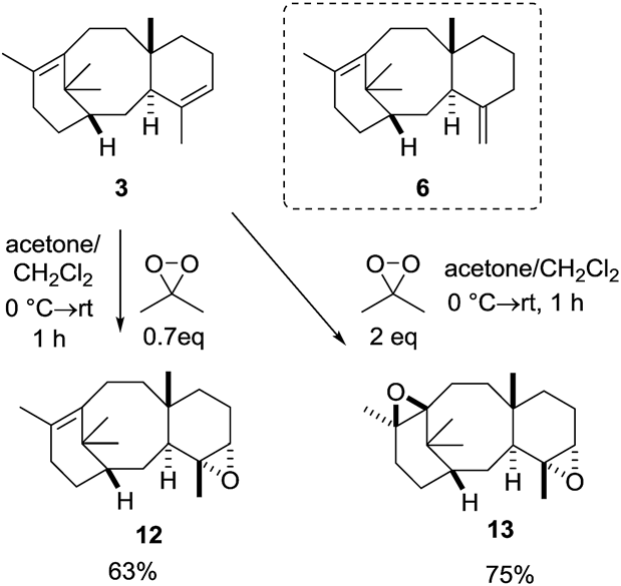
DMDO epoxidation of taxa-4(5),11(12)-diene.

Our studies began by isolating taxadiene from our previously described taxadiene synthase-containing tomatoes,^9^ using a slightly modified protocol that allows extraction directly from fresh fruit (see ESI† for details). This procedure afforded taxa-4(5),11(12)-diene (**3**) and taxa-4(20),11(12)-diene (**6**) as an inseparable 17 : 1 (**3** : **6**) mixture. With ready access to taxadiene we next turned our attention to epoxidation of **3**, with DMDO being selected as the oxidant due to its ease of use.^12^ As we were concerned with the potential over-epoxidation of taxadiene, we performed the reactions with substoichiometric quantities of oxidant. Pleasingly, when taxa-4(5),11(12)-diene (**3**) was treated with 0.7 equivalents of DMDO, the desired epoxide **12** was obtained as the major new product (95% purity as judged by ^1^H NMR; see ESI†) and unreacted taxadiene was recovered (Scheme 5).

Whilst the epoxide derived from **6** was not observed, the recovered taxadiene (20%) was significantly enriched in **6** (1 : 2; **3** : **6**) compared to the starting material (17 : 1; **3** : **6**), thus indicating that **3** is much more reactive towards epoxidation than **6**. Care had to be taken during chromatography on silica gel as the epoxide **12** was acid sensitive (*vide infra*). Treatment of taxadiene (**3**) with excess DMDO (2 equivalents), produced the bis-epoxide **13** in 75% yield, and this epoxide was found to be much more stable than **12** to chromatography on silica gel (Scheme 5).

### Synthesis of taxa-4(20),11(12)-dien-5α-ol (**4**)

**Scheme 6:**
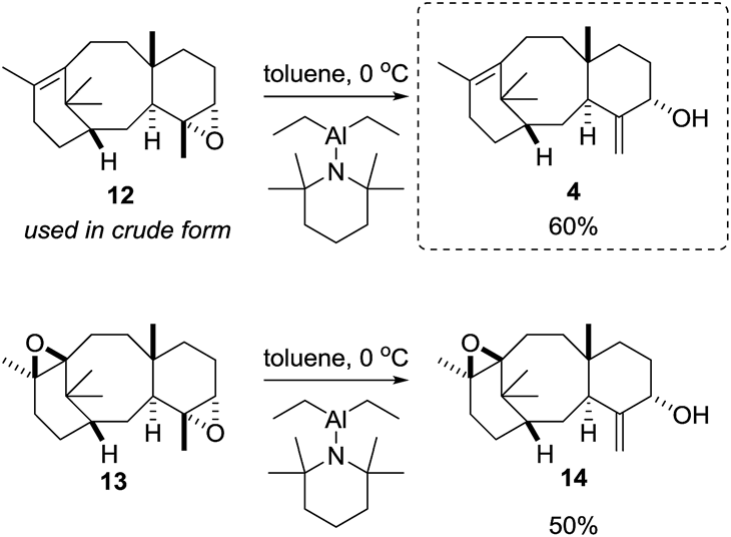
Synthesis of taxa-4(20),11(12)-dien-5α-ol (**4**) *via* rearrangement of epoxide **12**.

**Fig. 1:**
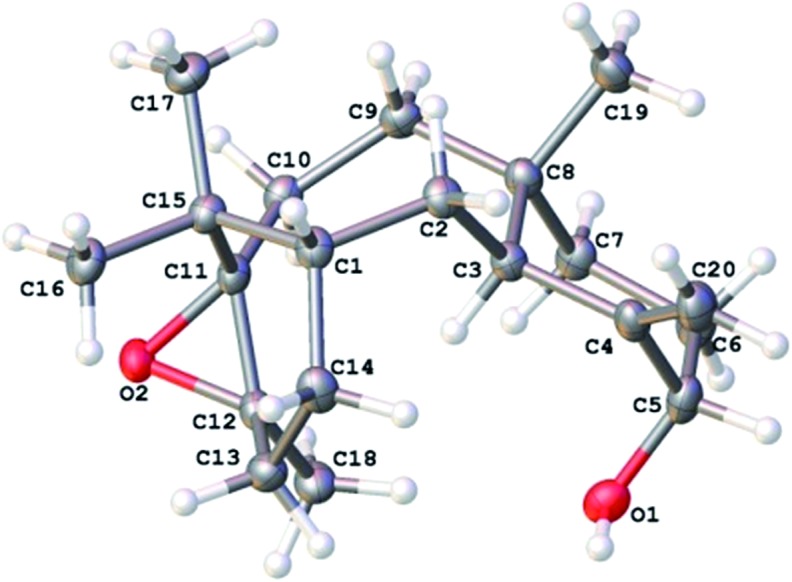
X-ray crystal structure of the taxadiene derived epoxyalcohol **14**.†

With a reliable route to the key epoxide **12** secured, we next wanted to assess its ability to act as a precursor to taxa-4(20),11(12)-dien-5α-ol (**4**). Before examining conditions of relevance to the biosynthesis, we first reacted **12** with Yamamoto's aluminium amide reagent (TMPAlEt_2_) to produce **4** as a reference sample (Scheme 6).^13^ As the epoxide **12** was prone to decomposition during column chromatography (*vide infra*), we used the epoxide in crude form directly from the DMDO oxidation. Thus, treatment of unpurified **12** with freshly prepared Yamamoto's reagent (BuLi, TMP, ClAlEt_2_, 0 °C, PhMe) gave taxa-4(20),11(12)-dien-5α-ol (**4**) in 60% isolated yield over the two steps from taxadiene (**3**). The spectroscopic data for **4** matched that reported by Williams for the 5α-stereoisomer,^5^ and this enabled us to confirm that epoxidation (**3** → **12**) must have occurred on the α-face of taxadiene. Having prepared bis-epoxide **13**, we next examined its behaviour under the same rearrangement conditions. Thus, treatment of **13** with Yamamoto's reagent provided epoxy-alcohol **14** as the major isolable product (50%). It is interesting to note that the 11(12)-epoxide moiety is also observed in natural taxanes such as taxinine A 11(12)-epoxide.^14^ Fortunately, **14** was obtained as a crystalline solid and we were able to determine an X-ray crystal structure (Fig. 1) to confirm the stereochemistry of the 11(12)-epoxide, and also show that the C5-hydroxyl was on the α-face.

### Rearrangements of taxadine-4(5)-epoxide **12**

Encouraged by the successful conversion of epoxide **12** to taxa-4(20),11(12)-dien-5α-ol (**4**) using Yamamoto's reagent, we next explored the behaviour of **12** under conditions of more relevance to the biosynthesis. We speculated that if taxadiene hydroxylase acts as a monooxygenase and epoxidises taxadiene **3** to produce **12**, then this would initially leave a mild Lewis acidic iron centre in close proximity to the epoxide, which could catalyse subsequent rearrangement reactions. Therefore, we decided to examine the behaviour of **12** under a range of acidic conditions.

**Scheme 7:**
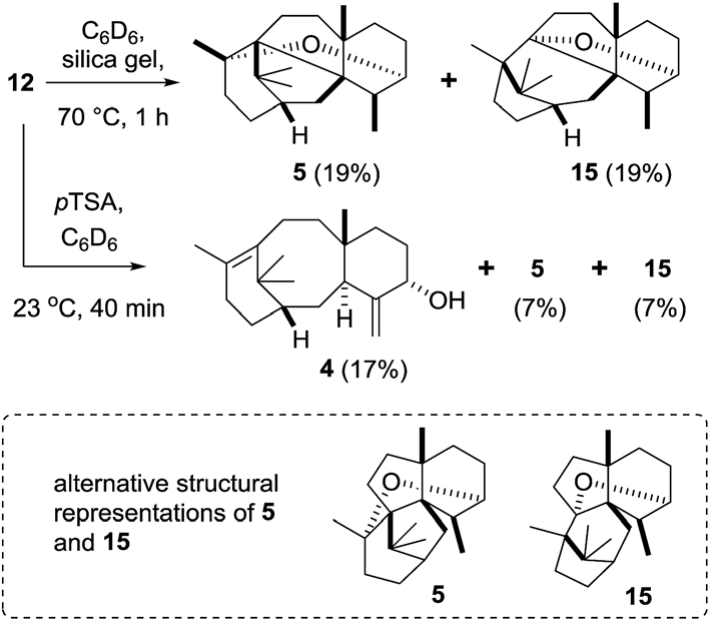
Rearrangement of taxadiene-4(5)-epoxide (**12**) under acidic conditions.

In order to simulate the acid-mediated decomposition encountered during silica gel chromatography, the epoxide **12** was treated with silica gel in C_6_D_6_ at 70 °C. Reaction progress was monitored by ^1^H NMR (see ESI†), and we determined that **12** converts into OCT (**5**), the molecule that had previously been produced in metabolically engineered tobacco by Rontein (Scheme 7),^7^ and the new isomeric oxacyclotaxane **15** (OCT2). Complete conversion of epoxide **12** was observed, as judged by the loss of the C19 methyl ^1^H NMR signal at 0.58 ppm, and the isomeric bridged ethers **5** and **15** were produced in an approximately 3 : 2 ratio (^1^H NMR). Chromatographic separation gave isolated samples of **5** (19%) and **15** (19%), which were then fully characterised.

**Scheme 8:**
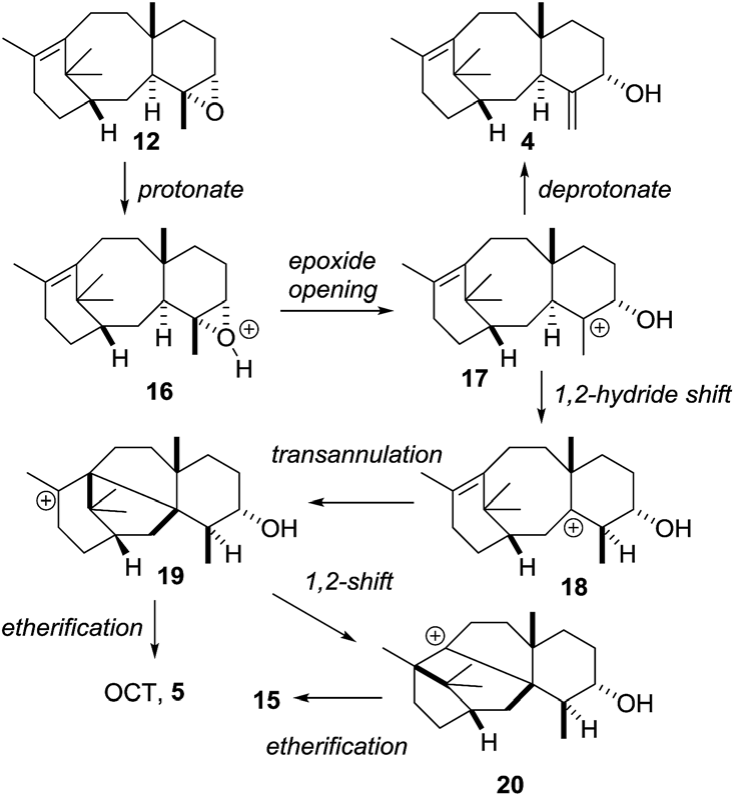
Proposed mechanisms for the formation of taxa-4(20),11(12)-dien-5α-ol (**4**), OCT (**5**) and OCT2 (**15**) from taxadiene-4(5)-epoxide (**12**).

Treatment of epoxide **12** with a stronger acid (*p*TSA, C_6_D_6_) gave taxa-4(20),11(12)-dien-5α-ol (**4**) as the major new product, with OCT (**5**) and OCT2 (**15**) being produced as minor products (isolated yields: **4** (17%); **5** (7%); **15** (7%)). The formation of 4(20),11(12)-dien-5α-ol (**4**) from the epoxide **12** under these strongly acidic conditions is readily explained by invoking protonation of the epoxide **12** to produce **16** (Scheme 8). Ring-opening then affords the cation **17**, and loss of a proton from the C20 methyl group installs the *exo*-methylene group in **4** (Scheme 8). The formation of OCT (**5**) also implicates the cation **17** as an intermediate. A 1,2-hydride shift first produces the new tertiary cation **18**, which next undergoes transannulation with the C11(12)-alkene leading to the cation **19**. Etherification, involving trapping the cation **19** with the secondary hydroxyl, then gives OCT (**5**). Similarly, the formation of OCT2 (**15**) can be rationalised by invoking a 1,2-alkyl shift of the tertiary cation **19**, leading to the new tertiary cation **20**, which is then trapped as the ether **15** by reacting with the C5-hydroxyl (Scheme 8).

As the biological oxidant (taxadiene hydroxylase^5^) acting upon taxadiene is a cytochrome P450, it is tempting to speculate that the reduced iron^III^ porphyrin (**11**, Scheme 4) is capable of facilitating a Lewis acid-catalysed rearrangement of the epoxide *in vivo*. Rontein, however, discounted this proposal^17^ on the basis that previous work on very different chemical systems has shown that iron^III^ porphyrins are poor catalysts for the rearrangement of epoxides.^15^ As we had access to the epoxide **12**, we could test this hypothesis experimentally, and we decided to treat **12** with an iron^III^ porphyrin.

**Scheme 9:**
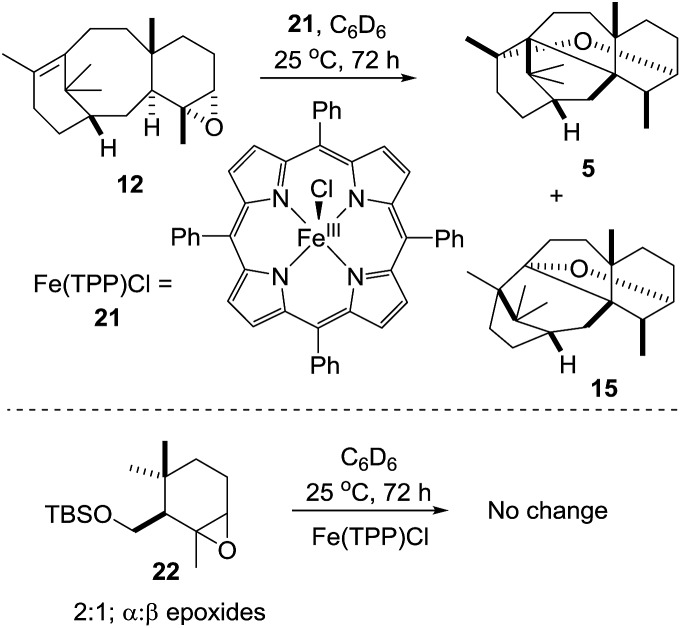
Iron^III^ porphyrin mediated rearrangement of taxadiene-4(5)-epoxide (**12**).

Contrary to the literature hypothesis, we were pleased to find that treatment of **12** with Fe^III^(TPP)Cl (2 equiv.) in C_6_D_6_ at 25 °C for 72 hours, lead to epoxide rearrangement, with the production of OCT (**5**) and OCT2 (**15**) as the main new products in a 1 : 1 ratio (^1^H NMR). Formation of taxa-4(20),11(12)-dien-5α-ol (**4**) was not observed under these Lewis acidic conditions (Scheme 9). As a control experiment, we exposed the similarly-substituted cyclogeraniol-derived epoxide **22**^16^ to the same Fe(TPP)Cl rearrangement conditions,^17^ and as expected from previous reports,^15^ no rearrangement was observed, thus highlighting the propensity of **12** to rearrange.

**Scheme 10:**
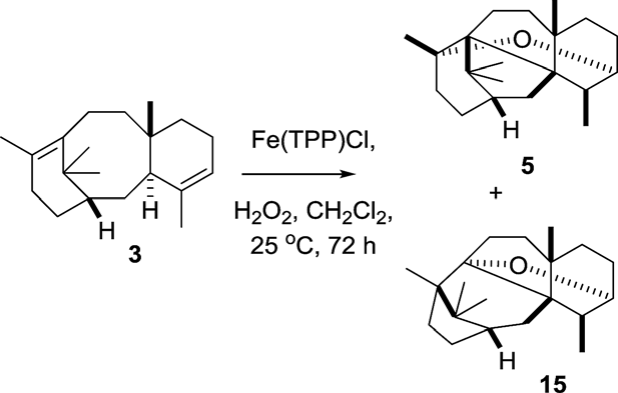
Iron^III^ porphyrin mediated oxidation of taxa-4(5),11(12)-diene (**3**).

Having shown that the two step epoxidation/Fe^III^ induced rearrangement mimics that seen *in vivo* (tobacco) mediated by taxa-4(5),11(12)-diene 5-hydroxylase (CYP725A4), we wondered if the initial oxidation of taxadiene could also be achieved using the Fe^III^(TPP)Cl catalyst and a suitable stoichiometric oxidant (Scheme 10). Thus, treatment of taxadiene (**3**) with Fe^III^(TPP)Cl (10 mol%) and hydrogen peroxide (1 equiv.)^18^ lead to complete consumption of starting material (as judged by t.l.c. and ^1^H NMR), and the subsequent production of oxidation products. Although the isolated yields were low, ^1^H NMR of the crude reaction mixture showed that the two major products were OCT (**5**) and the OCT2 (**15**). The production of taxa-4(20),11(12)-dien-5α-ol (**4**) was not observed under these conditions (Scheme 10).

### Implications for the taxol biosynthetic pathway

**Scheme 11:**
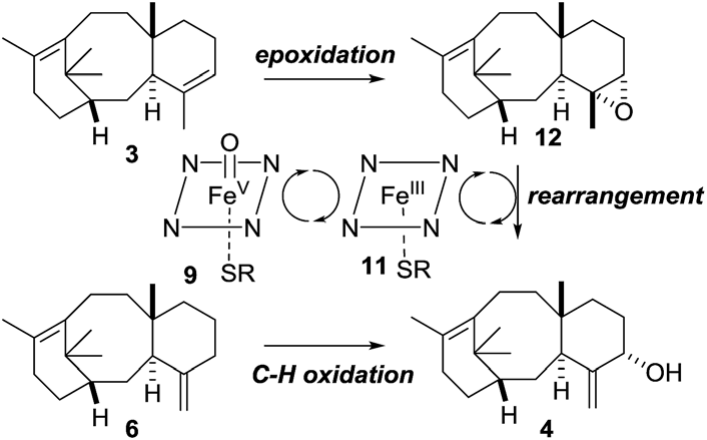
Proposal for the role of epoxide **12** in the biosynthesis of taxa-4(20),11(12)-dien-5α-ol (**4**).

As discussed in the introduction (Scheme 2), the current proposal for the biosynthesis of **4** from taxadiene **3** is that taxadiene hydroxylase performs an H-atom abstraction from the C20 methyl group of the 4(5)-alkene isomer of **3** to form the allyl radical **10**, and involvement of the epoxide **12** was rejected. Further support for the involvement of a common allyl radical **10** came from the fact that the 4(20)-alkene isomer **6** was also converted to taxa-4(20),11(12)-dien-5α-ol (**4**) by taxadiene hydroxylase. However, our experiments, coupled with the previously published kinetic isotope effect data,^5^ demonstrate that the epoxide **12** cannot be discounted as an intermediate on the taxol biosynthetic pathway. We have shown that the major, naturally occurring, 4(5)-alkene isomer of taxadiene **3** can be converted to taxa-4(20),11(12)-dien-5α-ol (**4**) *via* the epoxide **12**, and this suggests that the 4(5)-**3** and 4(20)-**6** alkene isomers of taxadiene are processed differently by taxadiene hydroxylase (Scheme 11).^19^

It is possible that 4(5)-alkene isomer **3** is epoxidised to produce **12**, which is then rearranged to **4**, by the action of the reduced form of the hydroxylase **11**. In contrast, the 4(20)-alkene isomer **6** could be converted directly to **4***via* the accepted H-atom abstraction mechanism. The involvement of epoxide **12** in the pathway provides an explanation for the lack of a significant primary kinetic isotope effect and the presence of an inverse secondary isotope effect when deuterium labelled [C20–^2^H_3_]-taxadiene (**7**) was oxidized by taxadiene hydroxylase. The labelled C20 methyl likely plays only a small role in the epoxidation process (*i.e.* leads to small inverse isotope effect), and loss of a proton from C20 in an intermediate such as **19** (Scheme 8) is unlikely to be rate-limiting.

## Conclusions

In this study, we have shown that taxa-4(5),11(12)-diene (**3**) can be isolated from the fruit of metabolically engineered tomatoes using our new optimised procedure. Furthermore, we have shown that taxadiene (**3**) can be epoxidised in a regio- and diastereoselective manner to provide taxadiene-4(5)-epoxide (**12**), and that this epoxide can be rearranged to give taxa-4(20),11(12)-dien-5α-ol (**4**) in 60% over the two chemical steps. We have shown that the epoxide **12** is sensitive to acids, and that both taxa-4(20),11(12)-dien-5α-ol (**4**), the known bridged ether OCT (**5**) and the new oxacyclotaxane (OCT2) **15** can be obtained from this material. We have shown that contrary to previous speculation, taxadiene-4(5)-epoxide (**12**) is susceptible to rearrangement when exposed to an iron^III^ porphyrin, and these observations combine to warrant reconsideration of the epoxide **12** as a chemically competent intermediate on the taxol biosynthetic pathway.

## Supplementary Material

Supplementary information

Crystal structure data
