# Supplementary material for: Accessing low-oxidation state taxanes: is taxadiene-4(5)-epoxide on the taxol biosynthetic pathway?
Source: Chem Sci. 2016 Jan 26;7(5):3102–7. doi: 10.1039/c5sc03463a (PMC6005263; doi:10.1039/c5sc03463a)

Electronic Supplementary Material (ESI) for Chemical Science.
This journal is © The Royal Society of Chemistry 2016

ELECTRONIC SUPPORTING INFORMATION

Accessing low-oxidation state taxanes: Is taxadiene-4(5)-epoxide on the taxol biosynthetic pathway?

Naomi A. Barton,[a] Benjamin J. Marsh,[a] William Lewis,[a] Nathalie Narraidoo,[b] Graham B. Seymour,[b] Rupert Fray[b] and Christopher J. Hayes[a]*

[a]*School of Chemistry, University of Nottingham, University Park, Nottingham, UK, NG7 2RD.*
[b]*Division of Plant and Crop Sciences, School of Biosciences, University of Nottingham, Sutton Bonington, Loughborough, UK, LE12 5RD*
Tel: +44 (0)115 951 3045; Fax: +44 (0)115 951 3564; e-mail: chris.hayes@nottingham.ac.uk

Table of Contents

**Page #**	**Experimental**	**Page #**	
1	Table of Contents	27	**15** (^{1}H)
2	General Details	28	**15** (^{13}C)
3	Experimental Details	29	**15** (HMBC)
		30	**15** (^{1}H COSY)
**Page #**	**NMR Spectra**	31	**15** (HSQC)
12	**3** (^{1}H)	32	**15 (**^{1}H NOSEY)
13	**3** (^{13}C)	33	**15** (NMR assignments)
14	**12** (^{1}H)	34	**15** (^{1}H COSY zoomed)
15	**12** (^{13}C)	35	**15** (HMBC zoomed)
16	**4** (^{1}H)	36	^{1}H NMR monitoring of rearrangement of **12** (pTSA)
17	**4** (^{13}C)	37	^{1}H NMR monitoring of rearrangement of **22** (Fe(TPP)Cl)
18	**13** (^{1}H)	38	^{1}H NMR monitoring of rearrangement of **12** (Fe(TPP)Cl)
19	**13** (^{13}C)	39	^{1}H NMR monitoring of oxidation of **12** (Fe(TPP)Cl + H_2O_2)
20	**14** (^{1}H)		
21	**13** (^{13}C)		
22	^{1}H NMR monitoring of rearrangement of **12** (silica gel)		
23	**5** (^{1}H)		
24	**5** (^{13}C)		
25	Comparison of NMR data for **5** to literature.		
26	**5** (^{1}H NOESY)		

General Details

Starting materials were obtained from suppliers and used without further purification unless stated otherwise. THF was distilled from potassium and benzophenone under a nitrogen atmosphere; dichloromethane was distilled from calcium hydride. Tetramethyl piperidine, and toluene were distilled from calcium hydride under an argon atmosphere. BF_3•$(OEt)_2$ was distilled under reduced pressure from calcium hydride and was then put under an argon atmosphere before use. ^{n}Butyl lithium and DMDO were titrated three times on the day of use. All water used was previously deionised and petrol refers to petroleum ether (b.p. 40 - 60 °C). Thin layer chromatography was carried out using Merck silica gel pre-coated sheets SIL G/UV_{254}, which were visualised under UV light before developing with either basic potassium permanganate solution or acidic solution of vanillin in ethanol. Column chromatography was carried out using Merck silica gel 60, 35-70 µm particles as the packing agent.

NMR spectra were obtained at 298 k as dilute solutions in deuterated solvent. The spectra were recorded on the δ scale in ppm and were referenced using the following: $CDCl_3$ δ_H 7.27, δ_C 77.1; C_6D_6, δ_H 7.15, δ_C 127.7. Spectra were recorded on Bruker DPX 400 MHz, AV 400 MHz, AV(III) 400 MHz or a AV(III) 500 MHz spectrometer. Assignments were made based on chemical shift with the aid of DEPT sequences and correlations techniques such as COSY, HMQC, HSQC, HMBC and NOSEY. The multiplicity of each signal is designated by the following abbreviations: s, singlet; d, doublet; t, triplet; q, quartet; app, apparent; br, broad or some combination thereof. *J* values are reported in Hertz (Hz) to the nearest decimal place and are rationalised. Relative stereochemistry was assigned using NOESY or nOe techniques.

Infrared spectra were recorded on a Perkin-Elmer 1600 FT spectrometer as dilute solutions in chloroform or as thin films on KBr discs. Mass spectra were recorded on a MicroTOF 61 spectrometer using electrospray or electron impact ionisation techniques with positive ion detection. Optical rotations ($[\alpha]_D$) were measured on a ADP440 polarimeter at a wavelength of 589 nm at a path length of 0.1 dm. Concentrations are given in g/100 mL.

Experimental Details

Isolation of taxa-4(5),11(12)-diene (3) and taxa-4(20),11(12)-diene (6)

Freshly picked taxadiene synthase-containing tomato fruit[19] (577 g) were crushed with a pestle and mortar. The resulting pulp was divided into three portions, and each was placed in a Büchner funnel and washed with acetone (3 x 75 mL). The acetone washings were combined and extracted with hexane (4 x 250 mL). The combined hexane extracts were dried ($MgSO_4$) and the solvent removed *in vacuo* to give an orange oil. Purification by column chromatography (eluting with 100% pentane) gave taxadiene (3 mg) as a colourless solid; R_f 0.60 (100% pentane). The combined, acetone-washed fruit pulp was transferred to a round bottomed flask and stirred with hexane (500 mL) for 3 days. The hexane was decanted and the solvent removed *in vacuo* to give an orange oil. Purification by column chromatography (eluting with 100% pentane) gave taxadiene (10 mg) as a colourless oil. Taxadiene was isolated as an inseparable 17:1 mixture of 4(5)- **3**:4(20)- **6** alkene isomers; R_f 0.60 (100% pentane); $[\alpha]^{23}_D$ = + 110 (*c* 0.8, $CHCl_3$) (lit: $[\alpha]^{20}_D$ = + 135 (*c* 0.32, $CHCl_3$); ν_{max} cm^{-1} ($CHCl_3$ soln) 2955, 2927, 2855, 1724, 1460, 1376, 1254, 1176, 1108, 1084, 1029, 967; ^1H NMR ($CDCl_3$, 400 MHz) (data for the major 4(5)- **3** isomer) 5.30 (1H, br s), 2.63 (1H, ddd, *J* 15.0, 10.5, 5.5), 2.53 (1H, br s), 3.37-2.27 (1H, m), 2.20-2.00 (3H, m), 1.93-1.79 (3H, m), 1.76-1.56 (5H, m), 1.70 (3H, br s), 1.67 (3H, s), 1.43 (1H, ddd, *J* 15.0, 6.5, 5.5), 1.34 (3H, s), 1.22-1.18 (1H, m), 1.04 (3H, s), 0.84 (3H, s); ^{13}C NMR (100 MHz, $CDCl_3$) (data for the major

4(5)- **3** isomer) 138.5 (C), 137.7 (C), 129.6 (C), 121.1 (CH), 44.3 (CH), 41.4 (CH_2), 39.8 (CH), 39.0 (C), 38.5 (CH_2), 37.3 (C), 30.7 (CH_3), 29.8 (CH_2), 28.4 (CH_2), 26.3 (CH_3), 24.5 (CH_2), 24.0 (CH_3), 23.2 (CH_2), 22.7 (CH_2), 21.7 (CH_3), 21.5 (CH_3); HRMS *m/z* (EI^+) 272.2498 (M^+, $C_{20}H_{32}^+$ requires 272.2499). The spectroscopic data for **3** are consistent with that reported previously.[3a,12,10]

Taxadiene-4(5)-epoxide (12)

Dimethyldioxirane[20] (1.12 mL of 41.3 mM solution in acetone, 46.3 µmol,) was added to a stirring solution of taxadiene (17:1; **3**:**6**) (20 mg, 73.5 µmol) in dichloromethane (1.5 mL) at 0 °C under argon and the resulting solution was stirred at ambient temperature for 1 h. The reaction was then concentrated *in vacuo*. Purification by column chromatography (ether:pentane; 1:9) afforded unreacted taxadiene (4 mg, 20%) in a 1:2 ratio of 4(5)- **3**:4(20)-**6** alkene isomers, R_f 0.95; (ether:pentane; 1:4). Further elution then afforded the epoxide **12** as a colourless solid (13 mg, 63%); R_f 0.59; (ether:pentane; 1:4) (**Please note**, the epoxide **12** is unstable on silica gel, and care must be taken to perform this purification as quickly as possible. In most circumstances, the crude epoxide can be used in subsequent reactions without purification); $[\alpha]^{23}_D$ = + 99 (*c* 0.4, C_6D_6); ν_{max} cm^{-1} ($CHCl_3$ soln) 3010, 2928, 2856, 1716, 1602, 1459, 1379, 1330, 1239, 1165, 1131, 1052, 1021, 965; ^1H NMR (500 MHz, C_6D_6) 2.69-2.62 (2H, m), 2.41 (1H, dd, *J* 6.0, 2.4), 2.30-2.23 (1H, m), 2.09-1.98 (3H, m), 1.95-1.86 (2H, m), 1.77-1.68 (1H, m), 1.74 (3H, s), 1.68-1.45 (5H, m), 1.29 (3H, s), 1.12 (3H, s), 1.09 (3H, s), 1.04-0.99 (1H, m), 0.84-0.80 (1H, m), 0.58 (3H, s); ^{13}C NMR (125 MHz, C_6D_6) 137.2 (C), 131.2 (C), 61.3 (CH), 60.6 (C), 44.3 (CH), 41.0 (CH), 40.3 (CH_2), 39.9 (C), 37.1 (C), 33.3 (CH_2), 31.6 (CH_3), 30.4 (CH_2), 28.2 (CH_2), 25.9 (CH_3), 25.3 (CH_2), 24.5 (CH_3), 23.7 (CH_2), 23.0 (CH_2), 22.8 (CH_3), 22.0 (CH_3); HRMS *m/z* (ESI^+) 289.2518 (M + H^+, $C_{20}H_{33}O$ requires 289.2526), 311.2341 (M + Na^+, $C_{20}H_{32}ONa$ requires 311.2345).

Taxadiene-4(5),11(12)-bisepoxide 13

Dimethyldioxirane[20] (890 µL of a 49.8 mM solution in acetone, 44.2 µmol,), was added to a stirring solution of taxadiene (17:1; **3**:**6**) (6 mg, 0.0221 mmol) in anhydrous dichloromethane (110 µL) at 0 °C under argon and the resulting solution was stirred at ambient temperature for 1 h. TLC analysis showed the presence of monoepoxide **12**, so an additional portion of dimethyldioxirane (220 µL of a 49.8 mM solution in acetone, 11.0 µmol), was added and the reaction stirred at ambient temperature for a further 0.5 h. The reaction was then concentrated *in vacuo*. Purification by column chromatography (ether:pentane; 1:9) afforded bis-epoxide **13** (5 mg; 75%); R_f 0.22 (ether:petrol; 1:4); ν_{max} cm^{-1} ($CHCl_3$ soln) 3008, 2960, 2930, 2877, 1482, 1460, 1381, 1354, 1306, 1287, 1263, 1242, 1178, 1136, 1097, 1070, 1055, 1040, 1021, 999, 965, 936, 909, 888; $[\alpha]^{23}_D = + 8.4$ (*c* 0.7, $CHCl_3$); ^1H NMR (400 MHz, $CDCl_3$) 2.96 (1H, dd, *J* 2.5, 0.5), 2.16-2.02 (3H, m), 1.98-1.89 (5H, m), 1.76 (1H, td, *J* 12.5, 7.0), 1.68-1.55 (3H, m), 1.61-1.58 (1H, m), 1.50 (3H, s), 1.49-1.42 (1H, m), 1.40 (3H, s), 1.31 (3H, s), 1.29-1.23 (1H, m), 0.96-0.87 (1H, m), 0.89 (3H, s), 0.79 (3H, s); ^{13}C NMR (100 MHz, $CDCl_3$) 65.4 (C), 62.2 (C), 61.1 (CH), 60.7 (C), 41.1 (CH), 40.0 (CH), 39.1 (C), 38.4 (CH_2), 35.5 (C), 31.3 (CH_2), 30.3 (CH_3), 26.8 (CH_2), 25.9 (CH_2), 25.7 (CH_2), 24.7 (CH_3), 24.6 (CH_3), 23.8 (CH_3), 22.1 (CH_2), 21.9 (CH_2), 21.5 (CH_3); HRMS *m/z* (ESI^+) 327.2293 (M + Na^+, $C_{20}H_{32}O_2Na$ requires 327.2295).

Taxa-4(20),11(12)-dien-5α-ol 4

A stock solution of Yamamoto's reagent was prepared as follows; ^nBuLi (2.50 mL, 5.0 mmol, 2.0 M in hexanes) was added to a stirring solution of tetramethylpiperidine (840 µL, 5.0 mmol, freshly distilled from CaH_2) and anhydrous toluene (5.0 mL) at 0 °C under argon and the resulting solution stirred for 40 min at 0 °C. Diethylaluminiumchloride (5.0 mL, 5.0 mmol, 1 M in heptane) was added to the solution at 0 °C and the resulting solution stirred at 0

°C for a further 40 min. A portion of the stock solution (277 μL, 0.104 mmol) was taken and added to a solution of crude monoepoxide **12** (10 mg, 34.7 μmol) in toluene (200 μL) at 0 °C under argon, and stirred at 0 °C for 1.5 h. The reaction was then quenched with saturated aqueous sodium hydrogen carbonate solution (10 mL). The aqueous layer was extracted with ether (3 x 10 mL) and then the combined organic extractions washed with brine (1 x 10 mL). The organic layer was then dried ($MgSO_4$) and concentrated *in vacuo*. Purification by column chromatography (ether:pentane; 1:9; rising to ether:pentane; 1:4) afforded taxadiene (2 mg; 20%). Further elution afforded taxa-4(20),11(12)-dien-5α-ol **4** as a colourless solid (6 mg; 60%); R_f 0.18 (ether:pentane; 1:9); $[\alpha]^{21}_D$ = + 108 (*c* 0.5, $CHCl_3$); ν_{max} cm^{-1} ($CHCl_3$ soln) 3603, 3079, 3055, 3008, 2982, 2930, 1643, 1601, 1461, 1378, 1296, 1240, 1125, 1107, 1054, 1018, 999, 986, 965, 941, 924, 902; ^1H NMR (400 MHz, $CDCl_3$) 4.94 (1H, dd, *J* 1.5, 1.0), 4.65 (1H, dd, *J* 1.5, 1.0), 4.26 (1H, t, *J* 3.0), 3.32 (1H, ddt, *J* 5.5, 2.0, 1.5), 2.85 (1H, td, *J* 13.5, 5.0), 2.40-2.25 (2H, m), 2.25-1.97 (3H, m), 1.94-1.86 (1H, m) 1.84 (3H, s), 1.79-1.74 (3H, m), 1.65 (1H, ddd, *J* 15.5, 5.5, 2.0), 1.56 (1H, ddd, *J* 15.5, 5.0, 2.0), 1.35 (3H, s), 1.31-1.20 (3H, m), 1.05 (3H, s), 1.02-0.97 (1H, m), 0.62 (3H, s); ^{13}C NMR (100 MHz, $CDCl_3$) 155.9 (C), 136.8 (C), 130.6 (C), 108.7 (CH_2), 74.6 (CH), 43.5 (CH), 40.05 (C), 39.96 (CH_2), 39.2 (C), 35.4 (CH), 32.7 (CH_2), 30.8 (CH_3), 30.17 (CH_2), 30.12 (CH_2), 28.0 (CH_2), 25.5 (CH_3), 24.7 (CH_2), 22.9 (CH_2), 22.1 (CH_3), 21.3 (CH_3); HRMS *m/z* (ESI^+) 311.2336 (M + Na^+, $C_{20}H_{32}ONa$ requires 311.2345), 289.2521 (M + H^+, $C_{20}H_{33}O$ requires 289.2526). The spectroscopic data for **4** are consistent with that reported previously.[6,8]

(1R,3S,5S,8S,11S,12R)-5-hydroxyl-11,12-oxiranyl-4,8,12,15,15 pentamethyltricyclo-[9.3.1.03,8]pentadeca-4,20-ene (14)

A stock solution of Yamamoto's reagent was prepared as follows; ^nBuLi (50.3 mL of a 1.5 M solution in hexanes, 75.5 mmol,) was added to a stirring solution of tetramethylpiperidine

(12.7 mL, 75.5 mmol) and anhydrous toluene (100 mL) at 0 °C under argon and the resulting solution stirred for 40 min at 0 °C. Diethylaluminiumchloride (75.5 mL of a 1 M solution in hexanes, 75.5 mmol,) was added to the solution at 0 °C and the resulting solution stirred at 0 °C for a further 40 min. A portion of the stock solution (145 µL, 0.046 mmol) was taken and added to a stirring solution of bis epoxide **13** (7 mg, 0.023 mmol) in toluene (40 µL) at 0 °C under argon, and the resulting solution stirred at 0 °C for 1 h. The reaction was then quenched with saturated aqueous sodium hydrogen carbonate solution (1 mL) then water (10 mL). The aqueous layer was extracted with ether (3 x 10 mL) and then the combined organic extractions washed with brine (2 x 10 mL). The organic layer was then dried ($MgSO_4$) and concentrated *in vacuo*. Purification by column chromatography (ether:petrol; 1:4) afforded unreacted starting material **13** (3.3 mg), R_f 0.22; (ether:petrol; 1:4). Further elution with ether:petrol 1:1 gave the allylic alcohol **14** as a colourless solid (1.5 mg, 21%); R_f 0.27 (ether:petrol; 1:1); $[\alpha]^{20}_D$ = + 32 (*c* 0.15, $CHCl_3$); ν_{max} cm^{-1} ($CHCl_3$ soln) 3591, 2922, 1607, 1005; ^1H NMR (400 MHz, $CDCl_3$) 5.07 (1H, s), 4.79 (1H, s), 4.38 (1H, br s), 2.82 (1H, br s), 2.30-2.11 (2H, m), 2.01-1.75 (5H, m), 1.72 (3H, s), 1.70-1.60 (3H, m), 1.54-1.46 (2H, m), 1.43 (3H, s), 1.39-1.33 (1H, m), 1.32-1.24 (2H, m), 1.03-0.96 (1H, m), 0.89 (3H, s), 0.69 (3H, s); ^{13}C NMR (125 MHz, $CDCl_3$) 154.2 (C), 110.0 (CH_2), 74.5 (CH), 65.6 (C), 62.2 (C), 41.0 (CH), 39.3 (C), 39.2 (CH_2), 39.0 (C), 37.3 (CH), 32.0 (CH_2), 30.4 (CH_3), 29.9 (CH_2), 29.7 (CH_2), 26.6 (CH_2), 26.2 (CH_2), 25.7 (CH_2), 24.7 (CH_3), 23.2 (CH_3), 21.7 (CH_3); HRMS *m/z* (ESI^+) 327.2283 (M + Na^+, $C_{20}H_{32}O_2Na$ requires 327.2295).

Rearrangement of Taxadiene-4(5)-epoxide 12 with Silica Gel

Dimethyldioxirane (524 µL of a 42.0 mM solution in acetone, 22.0 µmol,), was added to a stirring solution of taxadiene (10 mg, 36.7 µmol) in *d6*-benzene (0.7 mL) at 8 °C under argon. After 10 min the reaction was allowed to warm to room temperature and stirred for 1

h. The reaction was then concentrated *in vacuo*. Analysis by ^{1}H NMR (C_6D_6) showed a 1:4 ratio of unreacted taxadiene:epoxide **12**. This crude mixture was dissolved in *d6*-benzene (0.7 ml) and heated to 70 ^{o}C for 1 h. After cooling to room temperature, analysis by ^{1}H NMR (C_6D_6) showed no decomposition of starting epoxide **12**. Silica (100 mg) was added and the reaction was heated at 70 ^{o}C for 1 h. After cooling to room temperature, the reaction mixture was filtered and analysis by ^{1}HNMR (C_6D_6) showed complete conversion to OCT **5** and OCT2 **15**. Concentration *in vacuo*, and purification by silica gel chromatography (pentane → ether:pentane; 1:9) afforded recovered taxadiene as a colourless oil (1.2 mg), followed by OCT2 **15** as a colourless oil (2 mg, 19%); R_f 0.75 (ether:pentane; 1:9); $[\alpha]^{20}_{D}$ = + 31.5 (*c* 0.09, C_6D_6); ^{1}H NMR (500 MHz, C_6D_6) 4.02 (1H, app. t, *J* 5.2), 2.36-2.26 (2H, m), 2.13 (1H, dd, *J* 10.2, 14.8), 2.02 (1H, ddd, *J* 8.7, 12.2, 14.8), 1.86-1.66 (5 H, m), 1.58-1.50 (2H, m), 1.42 (1H, ddd, *J* 4.5, 12.0, 14.3), 1.36-1.21 (4H, m), 1.16 (3H, s), 0.95 (3H, s), 0.93 (3H, s), 0.88 (3H, s), 0.86 (3H, d); ^{13}C NMR (125 MHz, C_6D_6) 96.9 (C), 76.4 (CH), 52.5 (C), 50.3 (C), 50.0 (C), 46.1 (CH), 44.0 (CH_2), 43.2 (C), 42.4 (CH), 37.7 (CH_2), 33.3 (CH_2), 33.3 (CH_2), 28.8 (CH_2), 28.0 (CH_2), 27.1 (CH_3), 26.8 (CH_2), 26.4 (CH_3), 19.8 (CH_3), 16.4 (CH_3), 9.1 (CH_3); HRMS *m/z* (ESI^{+}) 289.2526 (M + H^{+}, $C_{20}H_{33}O$ requires 289.2526). Further elution gave OCT **5** as a colourless oil (2 mg, 19%). R_f 0.33 (ether:pentane; 1:9); $[\alpha]^{21}_{D}$ = + 12.4 (*c* 0.8, $CHCl_3$); ν_{max} cm^{-1} ($CHCl_3$ soln) 3054, 2958, 2930, 1601, 1466, 1376, 1239, 1152, 1107, 1070, 1043, 1030, 1003, 856; ^{1}H NMR (400 MHz, $CDCl_3$) 3.98 (1H, dd, *J* 9.0, 3.5), 2.49 (1H, qd, *J* 7.0, 3.5), 2.25 (1H, dd, *J* 13.0, 5.0), 2.15-1.79 (8H, m), 1.77-1.73 (1H, m), 1.70-1.51 (2H, m), 1.41-1.33 (3H, m), 1.23 (3H, s), 1.15 (3H, d), 1.07 (3H, s), 1.05 (3H, s), 0.96 (3H, s); ^{13}C NMR (100 MHz, $CDCl_3$) 80.1 (C), 69.6 (CH), 65.7 (C), 53.1 (C), 47.8 (CH_2), 45.9 (C), 45.8 (CH), 42.6 (C), 38.9 (CH_2), 37.4 (CH_2), 37.0 (CH), 36.3 (CH_2), 30.1 (CH_3), 30.1 (CH_2), 28.7 (CH_3), 28.0 (CH_3), 28.0 (CH_2), 27.3 (CH_2), 26.7 (CH_3), 15.2 (CH_3); HRMS *m/z* (ESI^{+}) 311.2335 (M + Na^{+}, $C_{20}H_{32}ONa$ requires 311.2345), 289.2522 (M + H^{+},

$C_{20}H_{33}O$ requires 289.2526). The spectroscopic data for **5** are consistent with that reported previously.[17,18]

Rearrangement of Taxadiene-4(5)-epoxide 12 with pTSA

Dimethyldioxirane (1.36 mL of a 43.6 mM solution in acetone, 59.5 μmol) was added dropwise to a stirring solution of taxadiene **3** (27 mg, 99.1 μmol) in *d6*-benzene (1.89 mL) at 8 °C under argon. The reaction was stirred for 10 min, warmed to room temperature and stirred for 1 h. The reaction was concentrated in *vacuo*. Analysis by ^{1}H NMR (C_6D_6) showed a 1:3.5 ratio of unreacted taxadiene:epoxide **12**. This crude mixture was dissolved in *d6*-benzene (1.89 mL) and *p*-toluenesulphonic acid monohydrate (11 mg, 59.5 μmol) was added. The reaction was stirred under argon at ambient temperature for 40 min before dilution with ether (5 mL) followed by water (5 mL). The layers were separated and the aqueous layer extracted with ether (3 x 5 mL). The combined organic layers were washed with brine (2 x 5 mL), dried ($MgSO_4$) and concentrated in *vacuo*. Analysis by ^{1}H NMR (C_6D_6) showed complete conversion of epoxide **12**, and appearance of characteristic signals corresponding to OCT **5**, OCT2 **15** and taxa-4(20),11(12)-dien-5α-ol **4**. Purification by silica gel chromatography (pentane → ether:pentane; 1:9) afforded OCT2 **15** (2 mg, 7%) and OCT **5** (2 mg, 7%) as colourless oils. Further elution then gave taxa-4(20),11(12)-dien-5α-ol **4** (5 mg, 17%). The spectroscopic data for **4**, **5** and **15** matched that reported above.

Rearrangement of Taxadiene-4(5)-epoxide 12 with Fe(TPP)Cl

Dimethyldioxirane (361 μL of a 51.0 mM solution in acetone, 18.4 μmol) was added dropwise to a stirring solution of taxadiene **3** (10 mg, 36.7 μmol) in *d6*-benzene (0.7 mL) at 8 °C under argon. The reaction was stirred for 10 min, warmed to room temperature and stirred for 2 h. The reaction was then concentrated *in vacuo*. Analysis by ^{1}H NMR (C_6D_6) showed a

1:1.4 ratio of unreacted taxadiene:epoxide **12**. This crude mixture was dissolved in *d6*-benzene (0.7 mL) and tetraphenylporphyrin iron (III) chloride (26 mg, 36.7 μmol) added. The reaction was stirred under argon at ambient temperature for 3 days before dilution with ether (10 mL) followed by water (10 mL). The layers were separated and the aqueous layer extracted with ether (3 x 10 mL). The combined organic layers were washed with brine (2 x 10 mL), dried ($MgSO_4$) and concentrated in *vacuo*. Analysis by ^1H NMR (C_6D_6) showed complete conversion of epoxide **8**, and appearance of characteristic signals corresponding to OCT **5** and OCT2 **15** (1:1 by ^1H NMR).

Oxidation of Taxadiene with Fe(TPP)Cl and H_2O_2

Tetraphenylporphyrin iron (III) chloride (2.6 mg, 3.67 μmol) was added to a stirring solution of taxadiene (17:1; **3**:**6**) (10 mg, 36.7 μmol) in anhydrous dichloromethane (0.5 mL). Aqueous hydrogen peroxide (30% w/w, 3.7 μL, 36.7 μmol) was added and the reaction stirred for 3 days at ambient temperature under argon. Water (3 mL) was added to the reaction and the layers separated. The organic layer was dried ($MgSO_4$) and concentrated *in vacuo*. Analysis by ^1H NMR showed a complex mixture of products. Purification by silica gel chromatography (ether:pentane; 1:9) afforded OCT2 **15** as a colourless oil (0.3 mg, 3%). Further elution gave OCT **5** (0.5 mg, 5%). The spectroscopic data for **5** and **15** matched that reported above.

Control experiment for the Fe(TPP)Cl rearrangement of epoxide 12

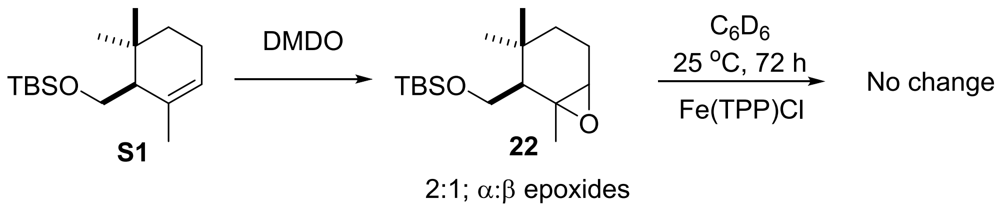

Dimethyldioxirane (363 μL, 18.6 μmol) was added dropwise to a stirring solution of alkene **S1**[25] (10 mg, 37.2 μmol) in dichloromethane (0.7 mL) at 0 °C under argon. The reaction was stirred for 10 min, warmed to room temperature and stirred for 1 h. Evaporation of solvent *in vacuo* and analysis by ^1H NMR showed a ratio of 1:0.7 (starting material: epoxide). The crude reaction mixture was dissolved in dichloromethane (0.7 mL), cooled to 0 °C, and a further portion of dimethyldioxirane (580 μL of a 51.3 mM solution in acetone, 29.8 μmol) was added dropwise. The reaction was warmed to room temperature, stirred for 20 min, and then concentrated *in vacuo* to give crude epoxide **22** (9 mg) as a 2:1 mixture of diastereoisomers. Epoxide **22** (9 mg) was dissolved in anhydrous dichloromethane (0.7 mL) and tetraphenylporphyrin iron (III) chloride (26 mg, 37.2 μmol) was added. The reaction was stirred under argon at ambient temperature for 3 days. The reaction was diluted with ether (5 mL), then water (5 mL), and the separated aqueous layer was extracted with ether (3 x 5 mL). The combined organic layers were then washed with brine (5 mL), dried ($MgSO_4$) and concentrated *in vacuo*. Analysis by ^1H NMR showed no change to the epoxide **22**.

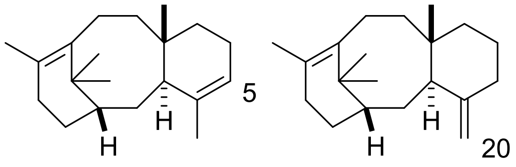
5
20
H
H
H
H

3 **6**

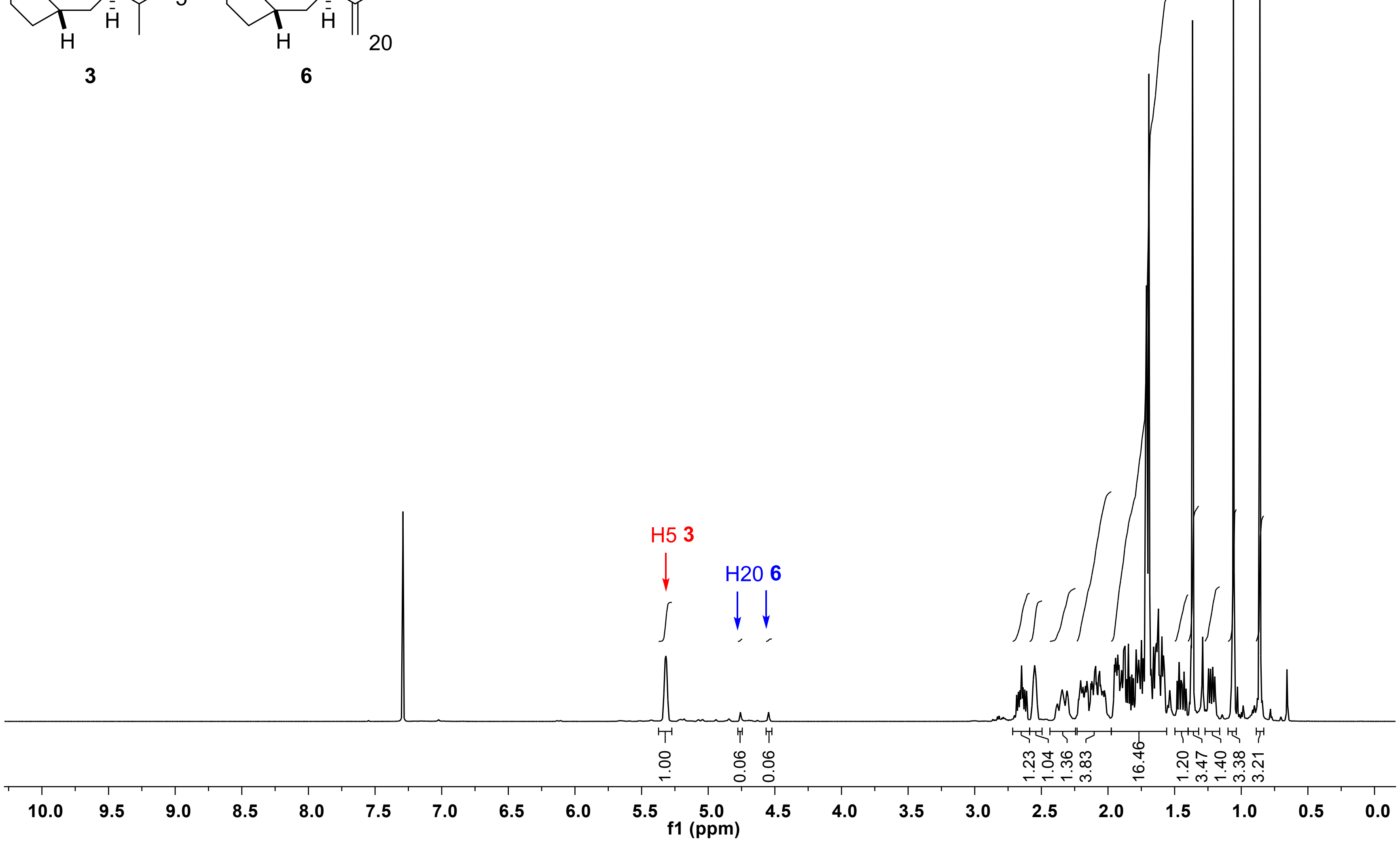
H5 3
H20 6
1.00
0.06
0.06
1.23
1.04
1.36
3.83
16.46
1.20
3.47
1.40
3.38
3.21
10.0
9.5
9.0
8.5
8.0
7.5
7.0
6.5
6.0
5.5
5.0
4.5
4.0
3.5
3.0
2.5
2.0
1.5
1.0
0.5
0.0
f1 (ppm)

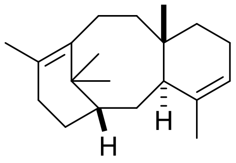

3

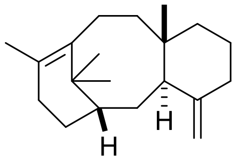

6

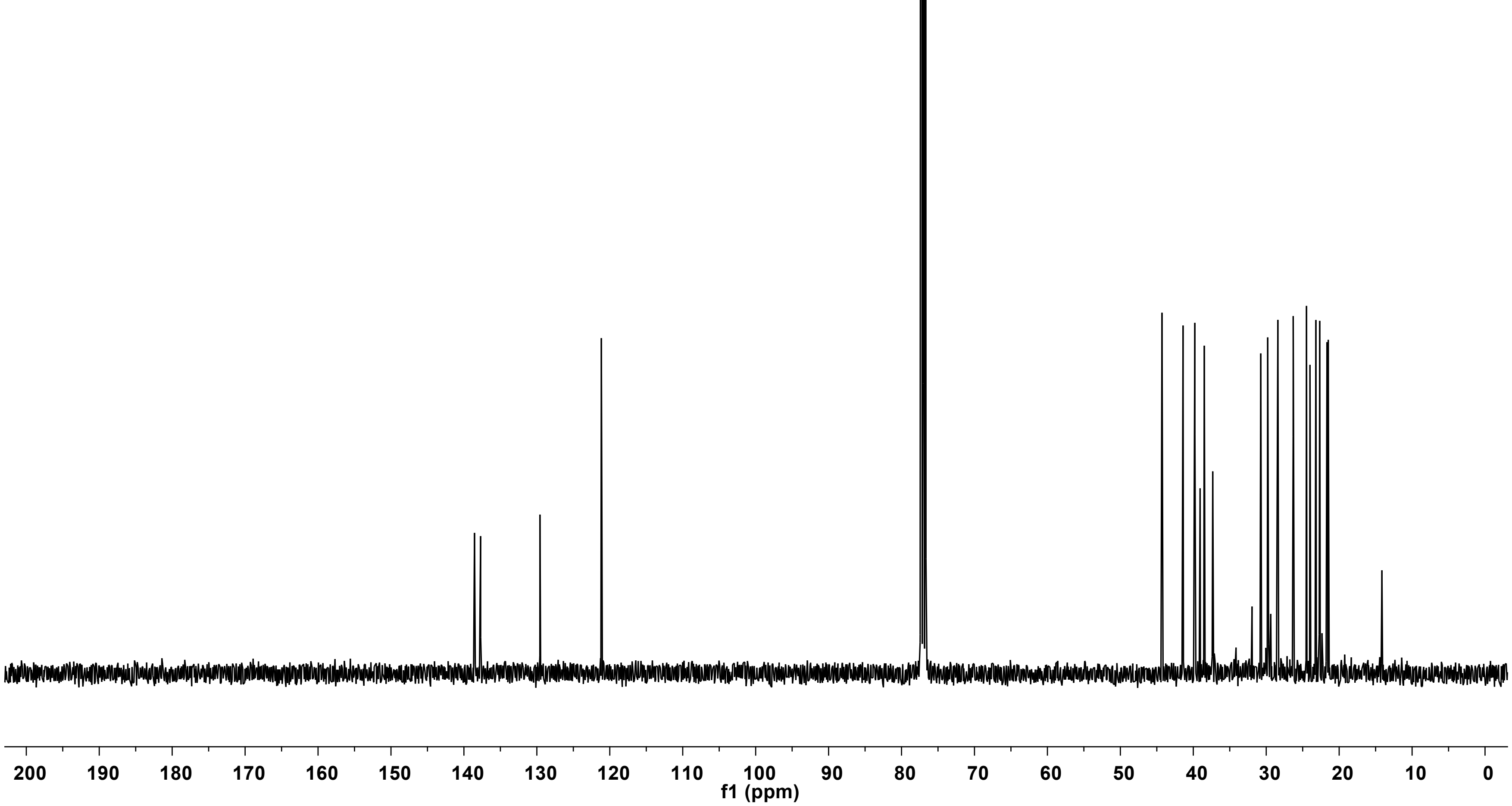
200
190
180
170
160
150
140
130
120
110
100
90
80
70
60
50
40
30
20
10
0
f1 (ppm)

^{1}H NMR (400 MHz, C_6D_6) – Taxa-4(5)-epoxide (**12**)

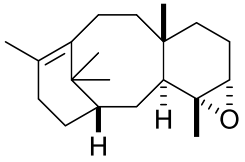

12

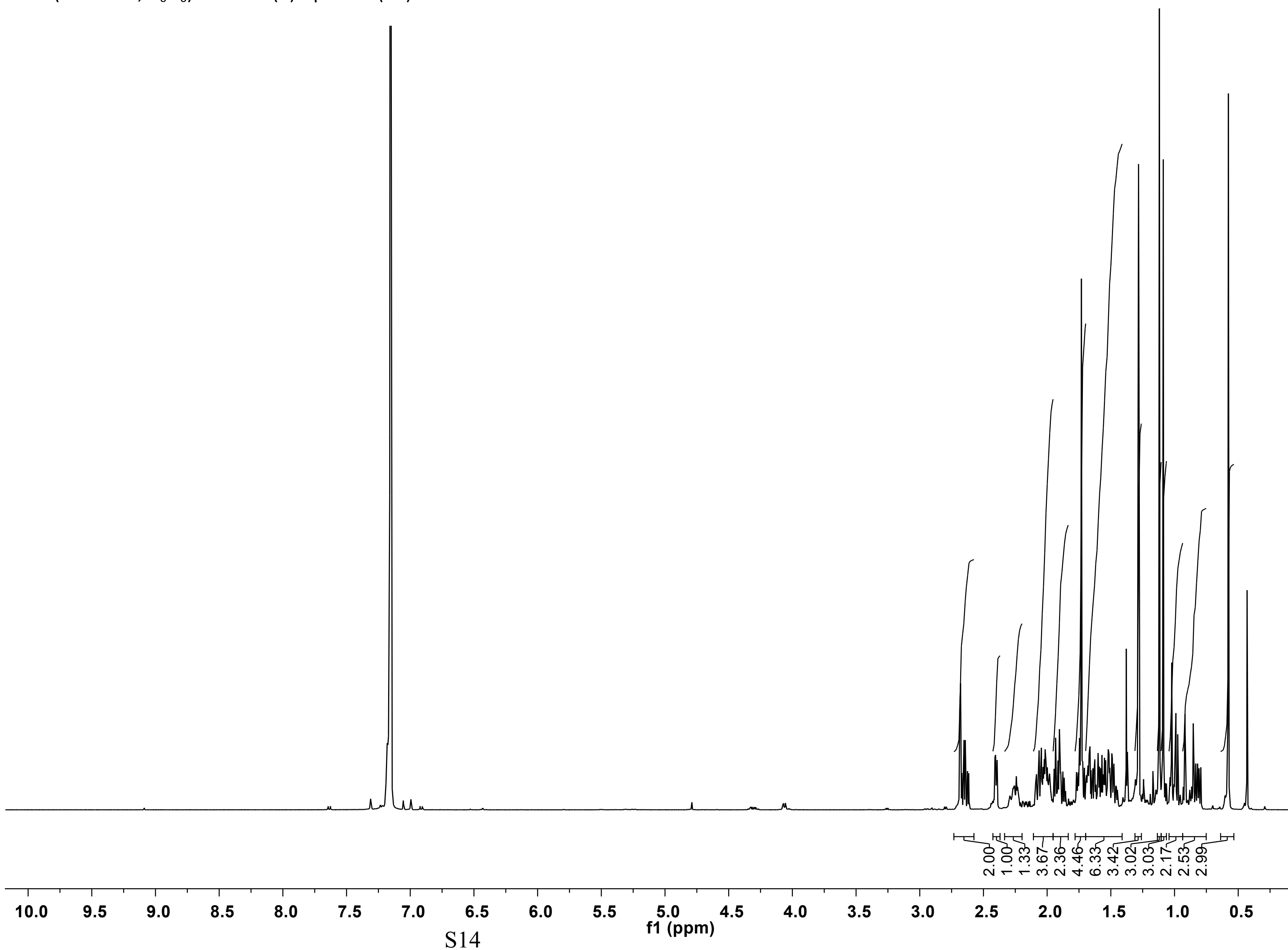

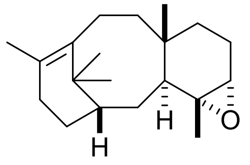

12

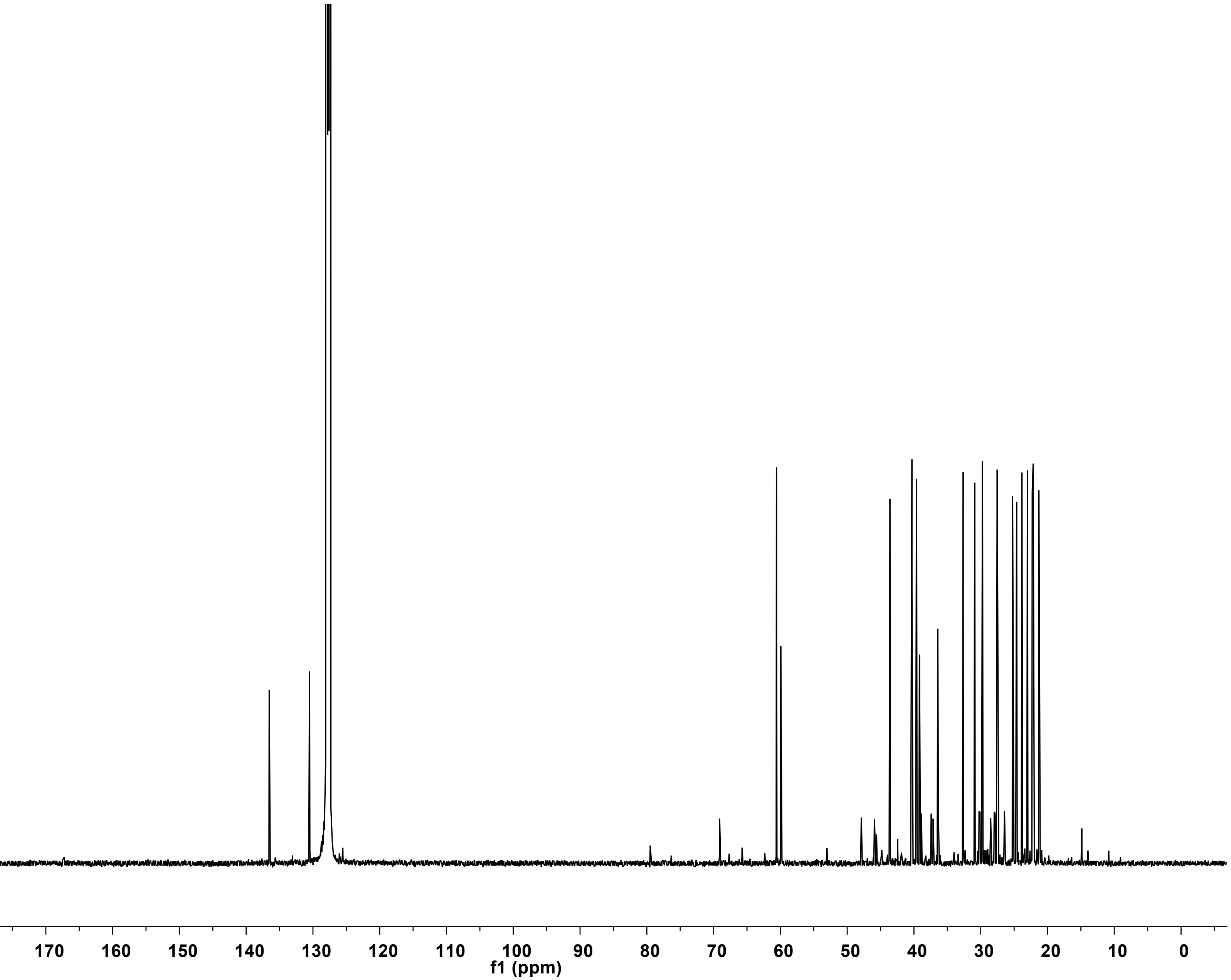
200
190
180
170
160
150
140
130
120
110
100
90
80
70
60
50
40
30
20
10
0
f1 (ppm)

^{1}H NMR (400 MHz, C

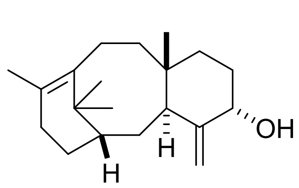

4

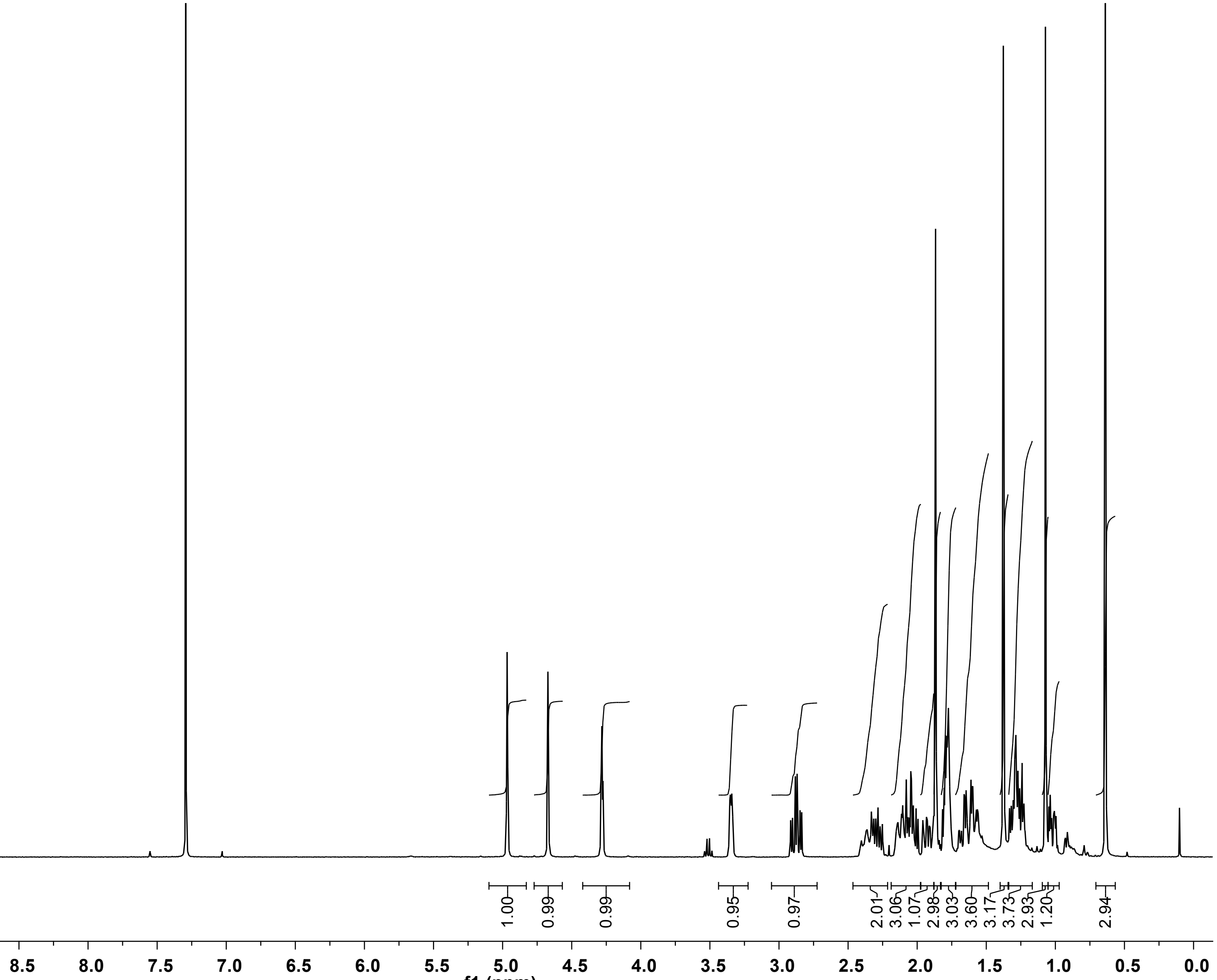

^{13}C NMR (100 MHz, $CDCl_3$) – Taxa-4(20), 11(12)-dien-5α-ol (**4**)

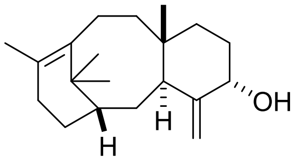

4

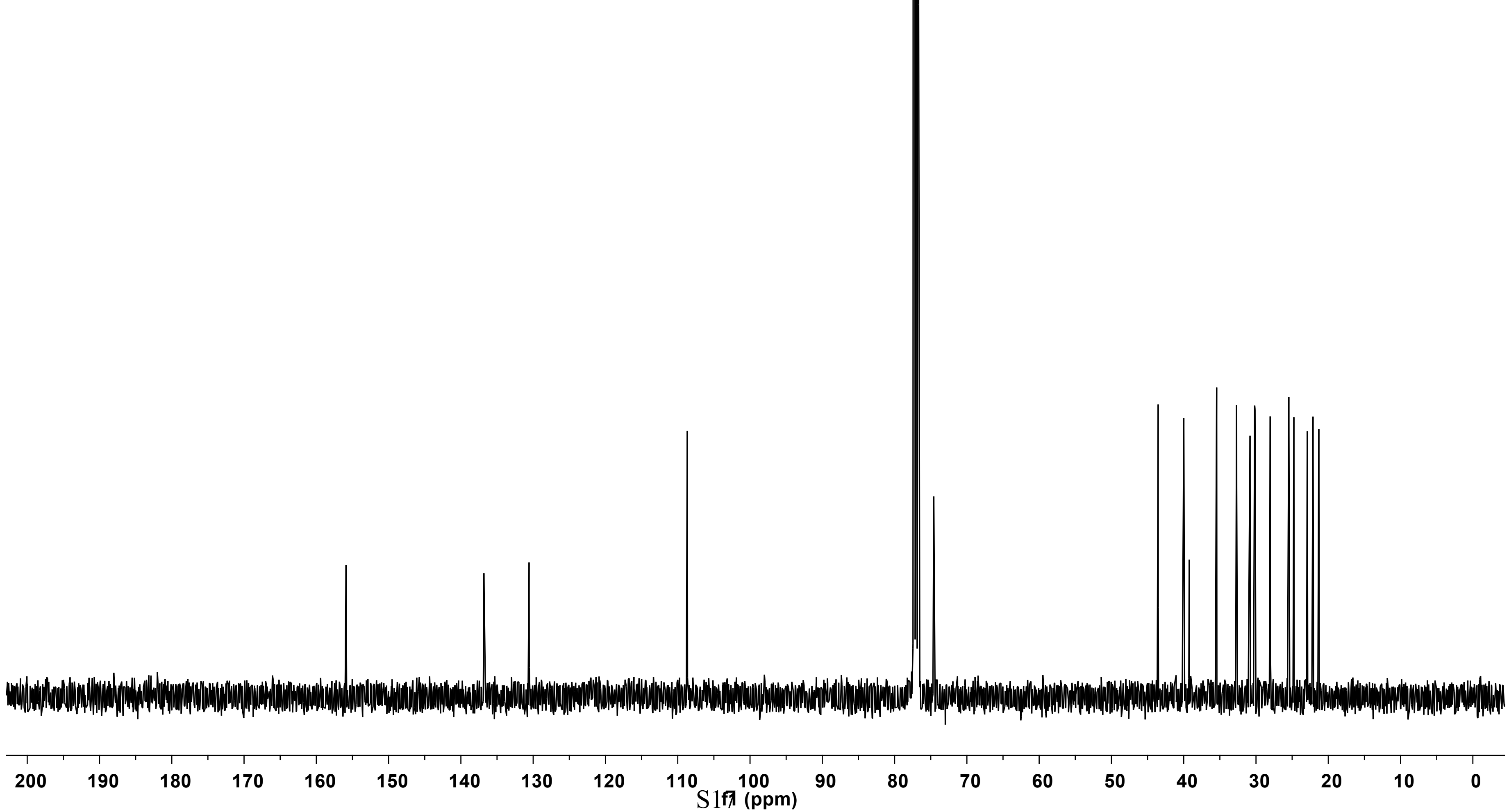

^{1}H NMR (400 MHz, $CDCl_3$) – bis epoxide (**13**)

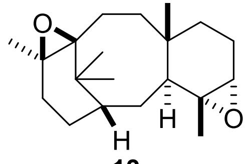

13

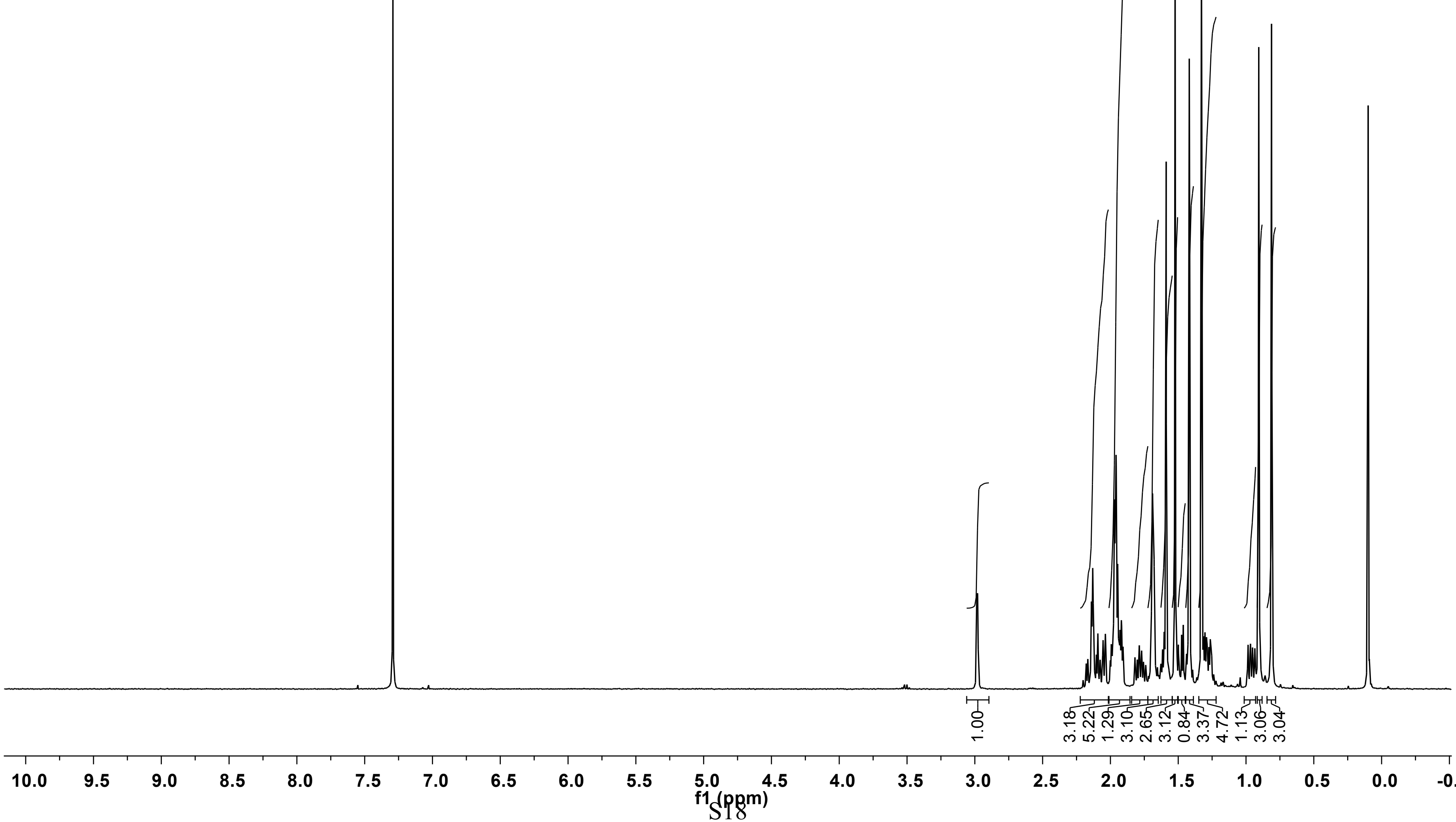

^{13}C NMR (100 MHz, $CDCl_3$) – bis-epoxide (**13**)

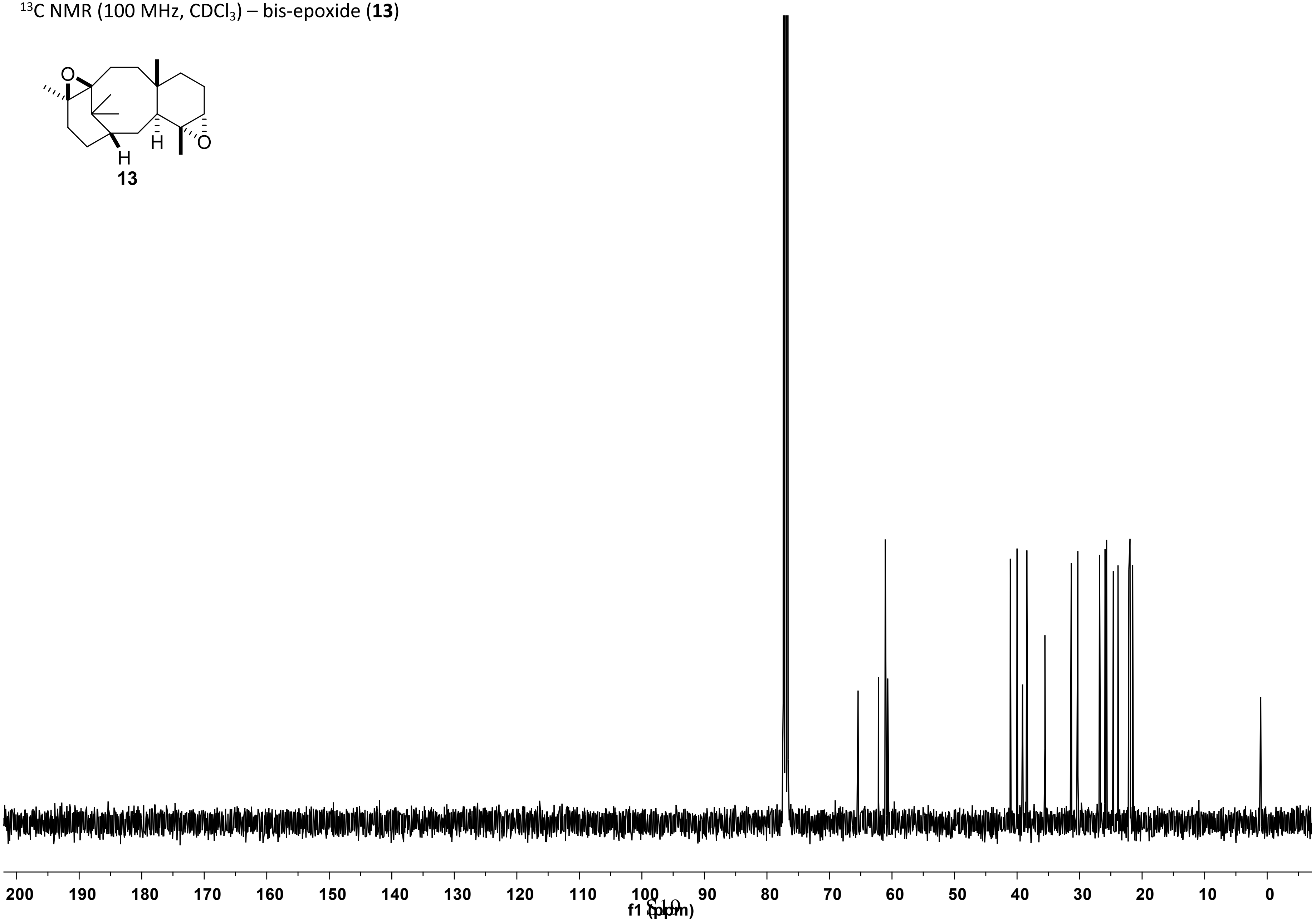

^{1}H NMR (400 MHz, $CDCl_3$) - Epoxy alcohol (**14**)

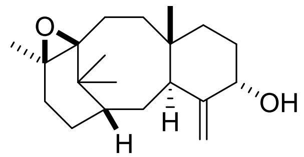

14

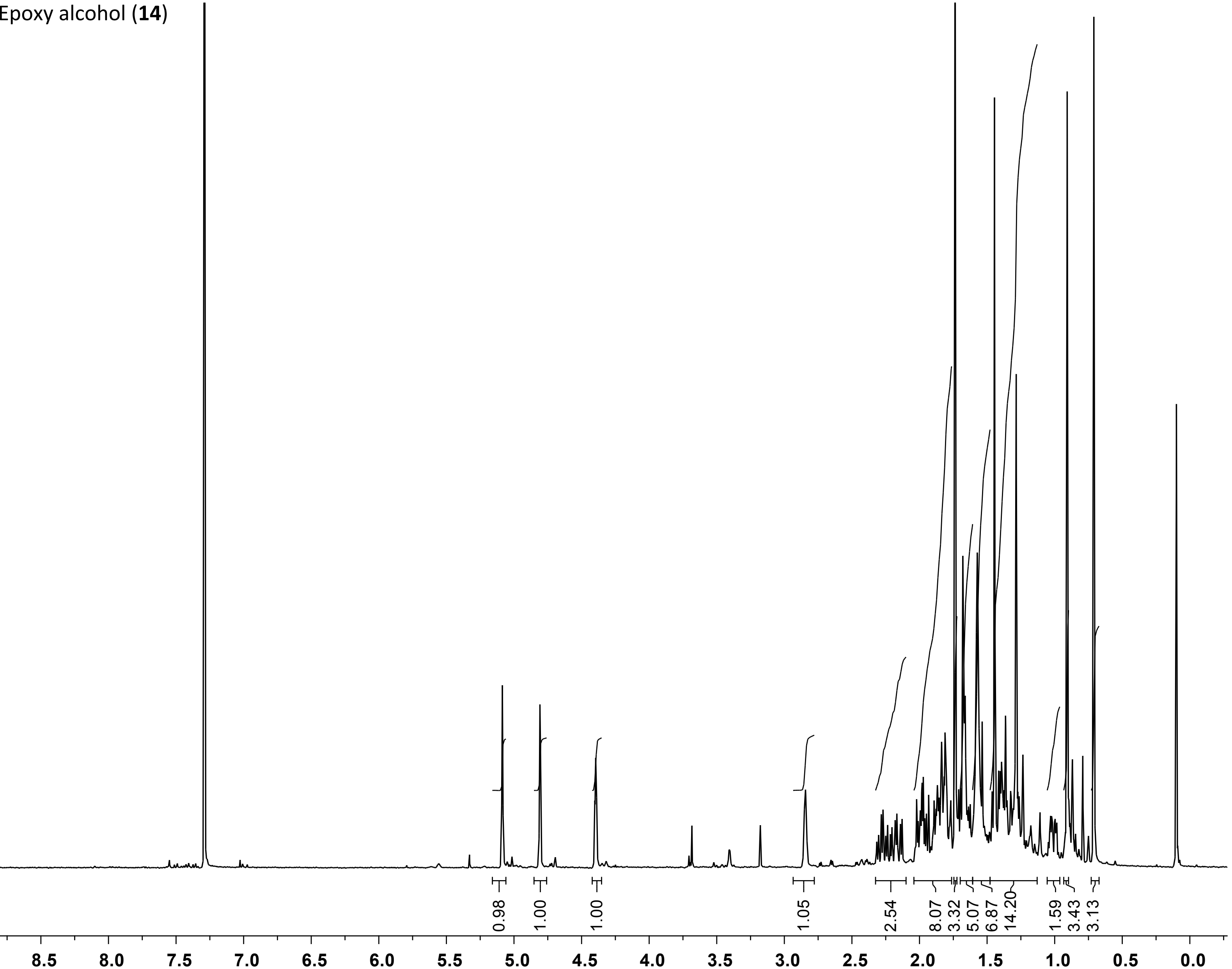

ELECTRONIC SUPPORTING INFORMATION

^{13}C NMR (100 MHz, $CDCl_3$) - Epoxy alcohol (**14**)

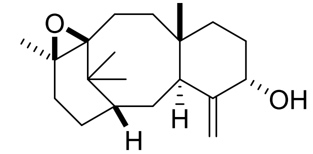

14

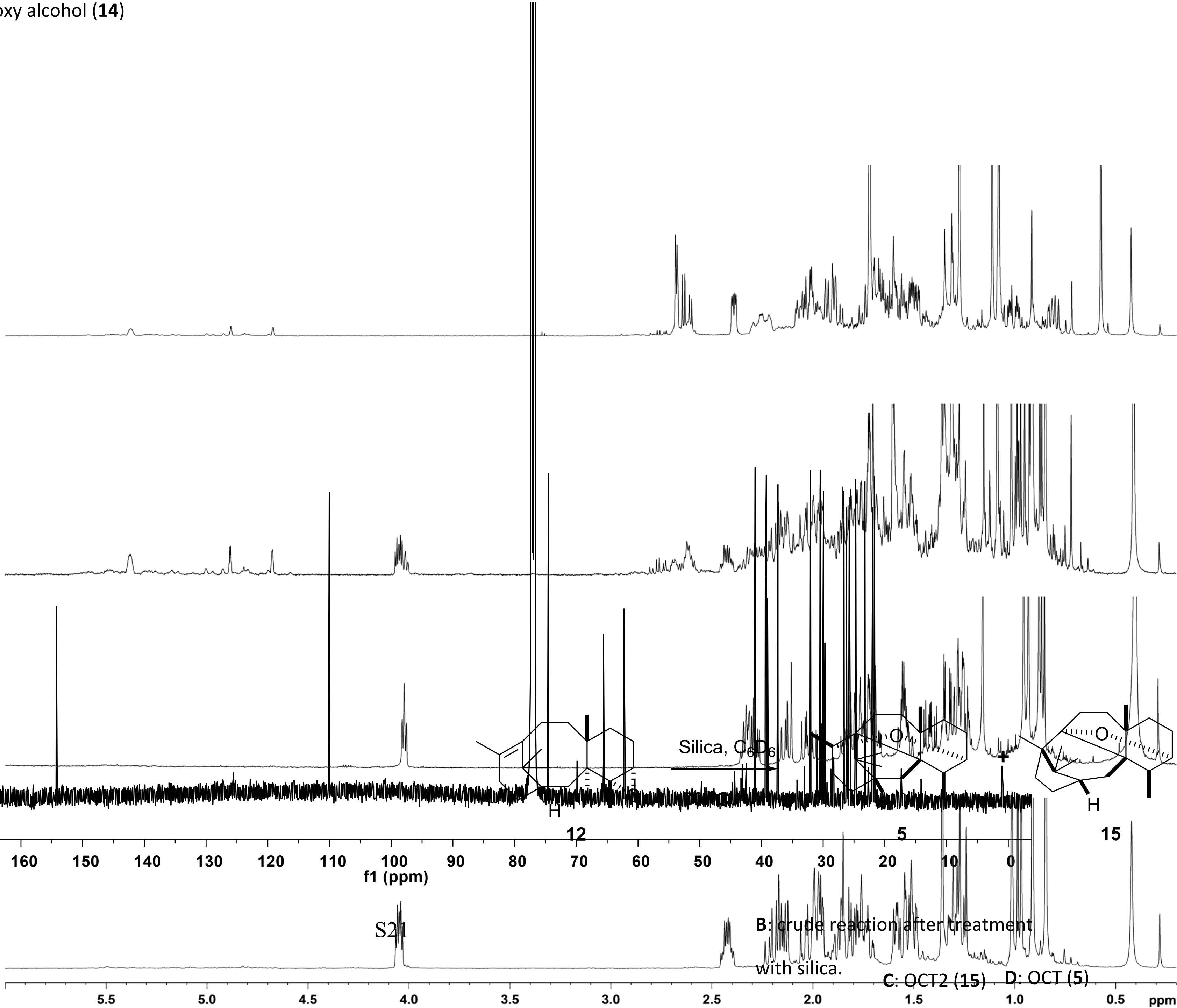

^{1}H NMRs (400 MHz, C_6D_6):

A: crude taxa-4(5)-epoxide (**12**) and remaining taxadiene (**3** and **6**)

^{1}H NMR (400 MHz, $CDCl_3$) – OCT (**5**)

IATION

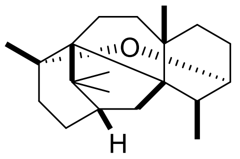

5

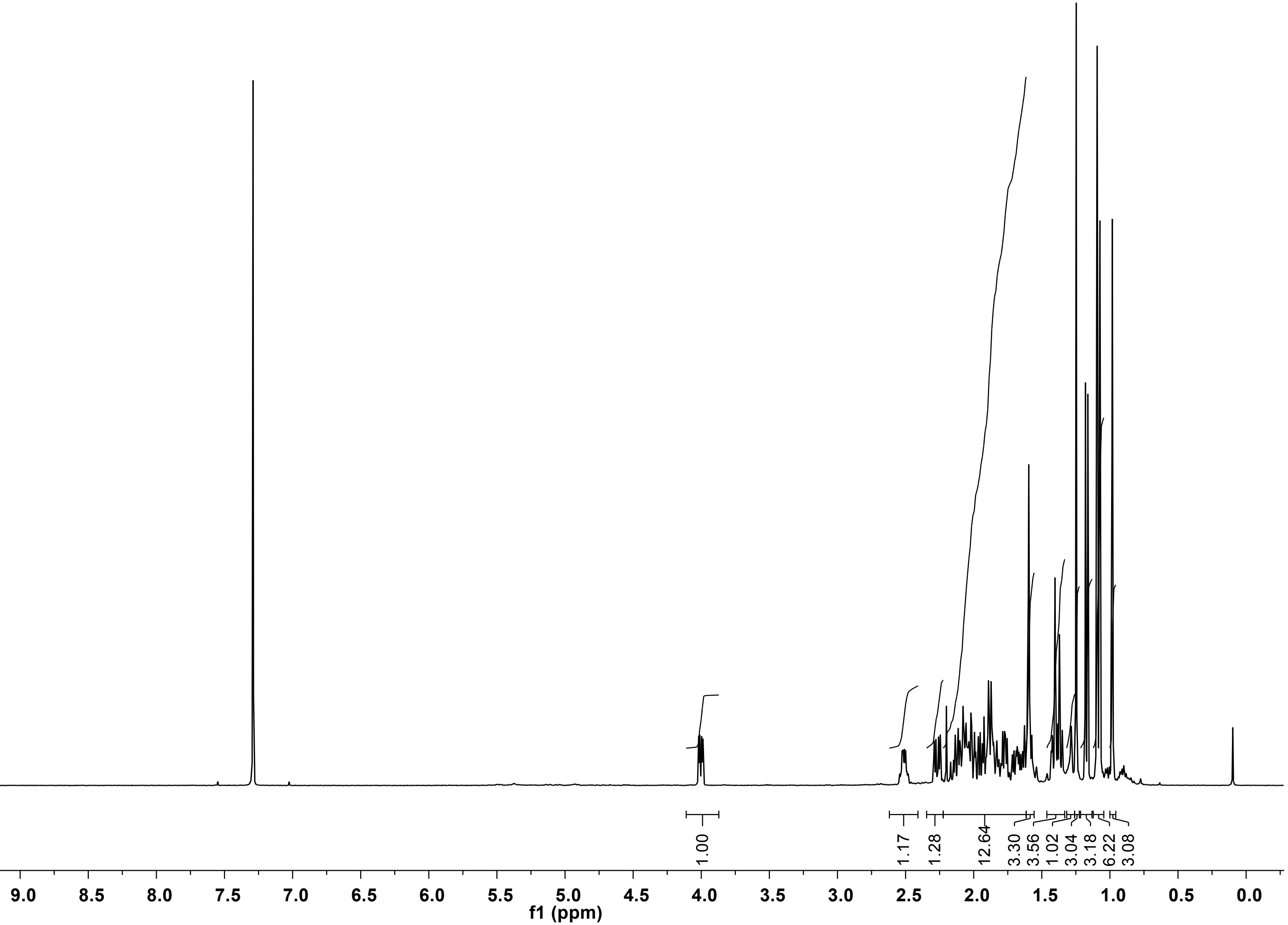

^{13}C NMR (125 MHz, $CDCl_3$) – OCT (**5**)

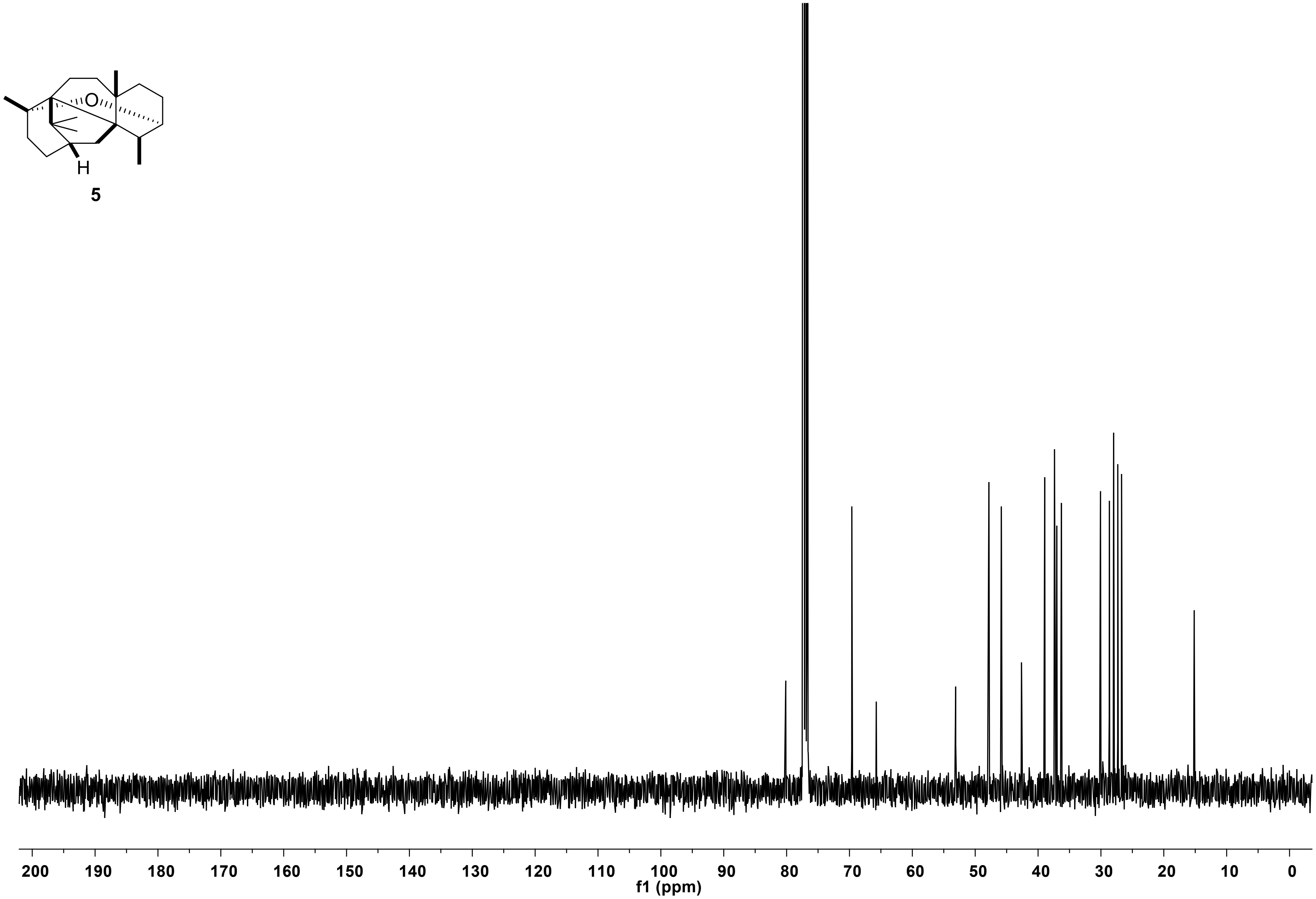

Table 1: Chemical shift comparison of our data for OCT **5** to that previously reported ($CDCl_3$)

Position	δ_H Literature	δ_H This Work	δ_c Literature	δ_c This Work
1	1.71	1.77-1.73	45.9	45.8
2	1.33	1.41-1.33	39.1	38.9
3			53.3	53.1
4	2.47	2.49	37.1	37.0
5	3.97	3.98	69.8	69.6
6	2.04	2.09-2.00	30.2	30.1
	1.82	1.87-1.81		
7	1.36	1.41-1.33	37.5	37.4
	1.83	1.87-1.81		
8			42.7	42.6
9	1.53	1.61-1.55	47.3	47.8
	1.82	1.87-1.77		
10	1.31	1.41-1.33	30.2	27.3
	1.38	2.15-2.05		
11			66.0	65.7
12			80.5	80.1
13	1.84	2.06-1.85	36.4	36.3
	1.98			
14	2.01	2.06-1.97	28.1	28.0
	1.62	1.70-1.61		
15			46.0	45.9
16	0.93	0.96	28.6	28.7
17	1.01	1.05	26.9	26.7
18	1.19	1.23	30.3	30.1
19	1.04	1.07	28.0	28.0
20	1.13	1.15	15.2	15.2

^{1}H NOESY – (500

Showing C-4 to C

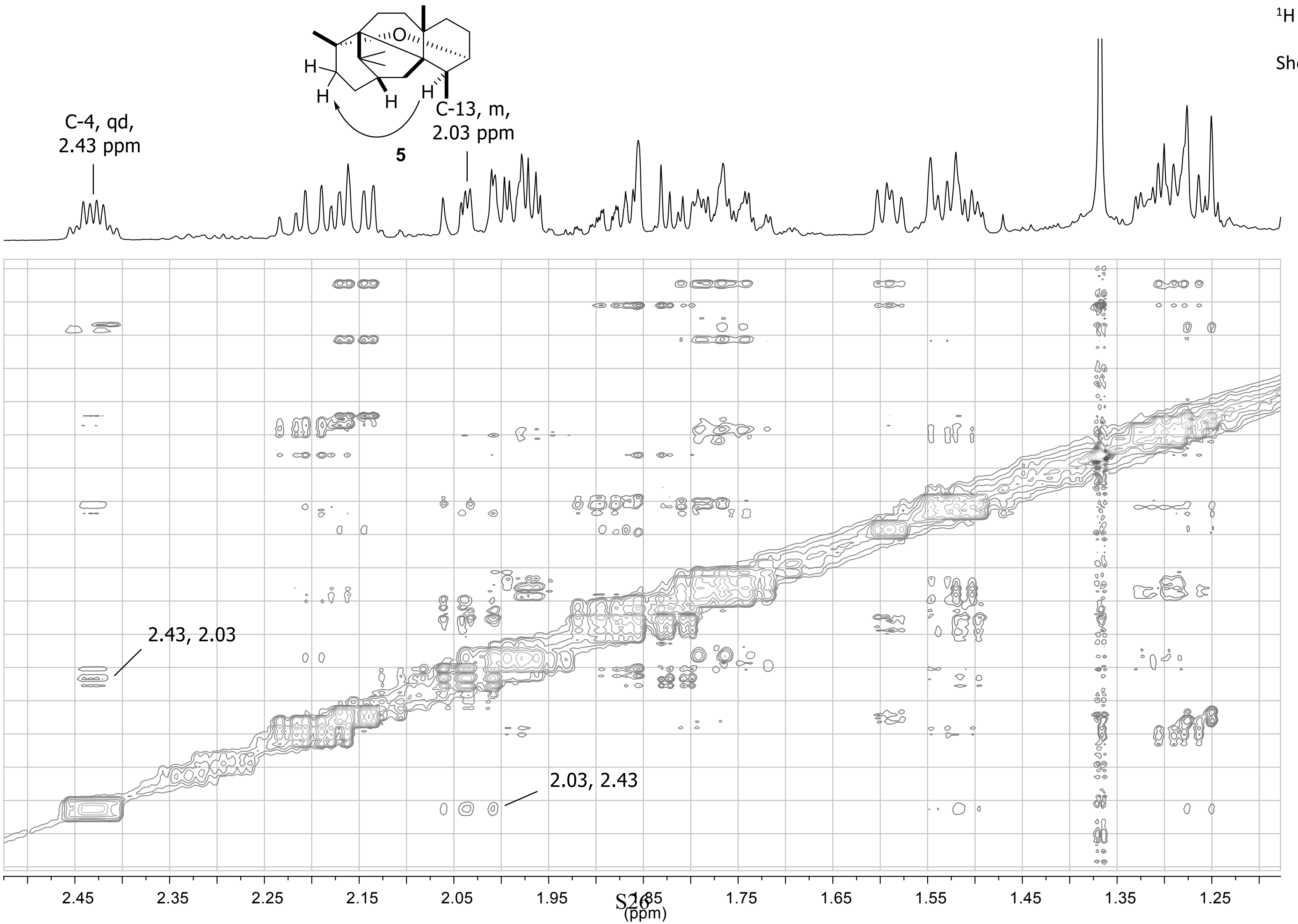

^1H NMR (500 MHz, C_6D_6) – OCT2 (**15**)

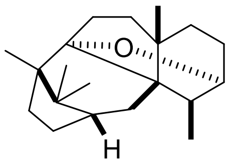

15

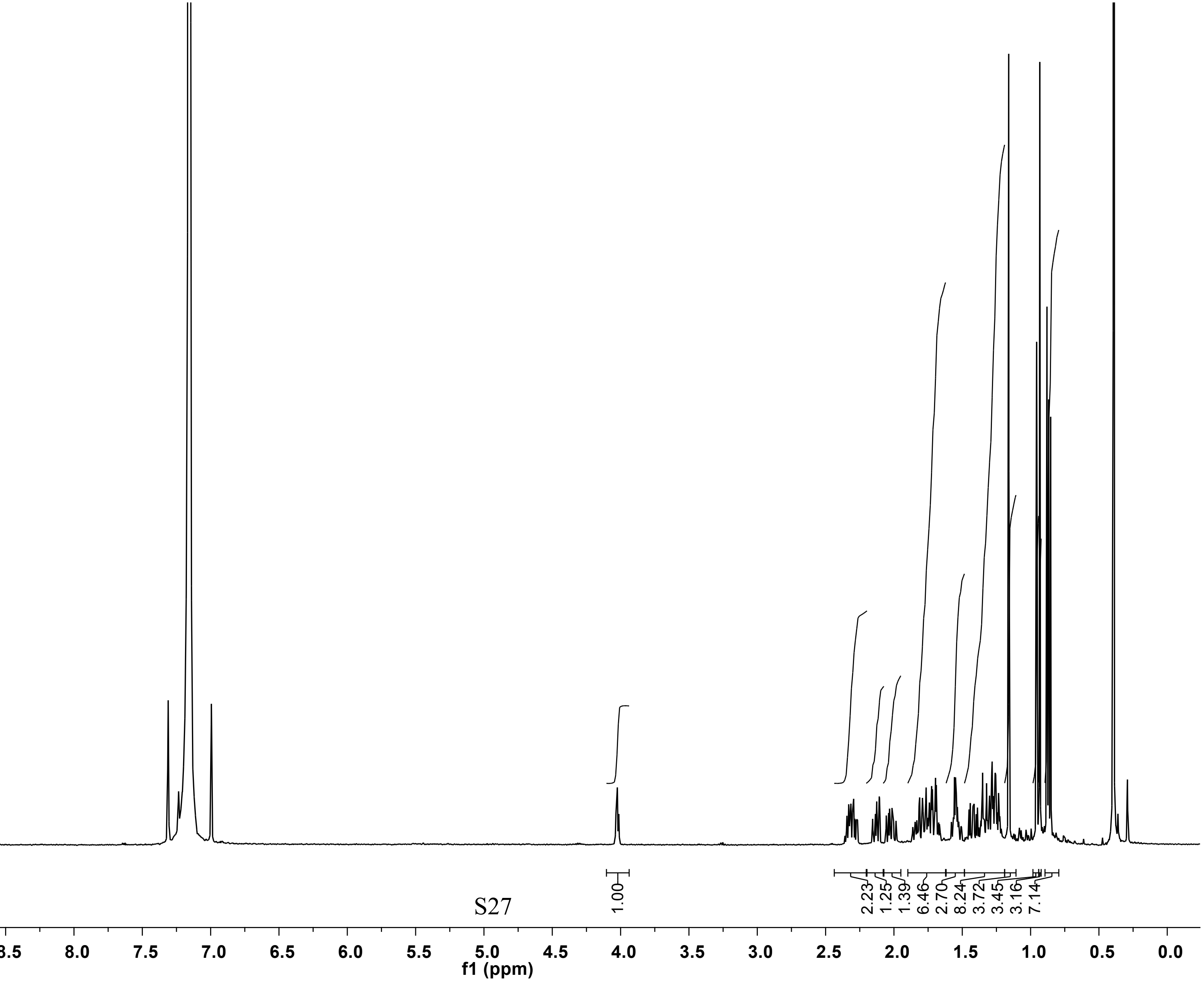

^{13}C NMR (125 MHz, C_6D_6) – OCT2 (**15**)

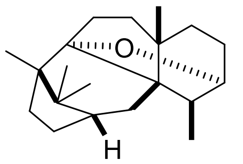

15

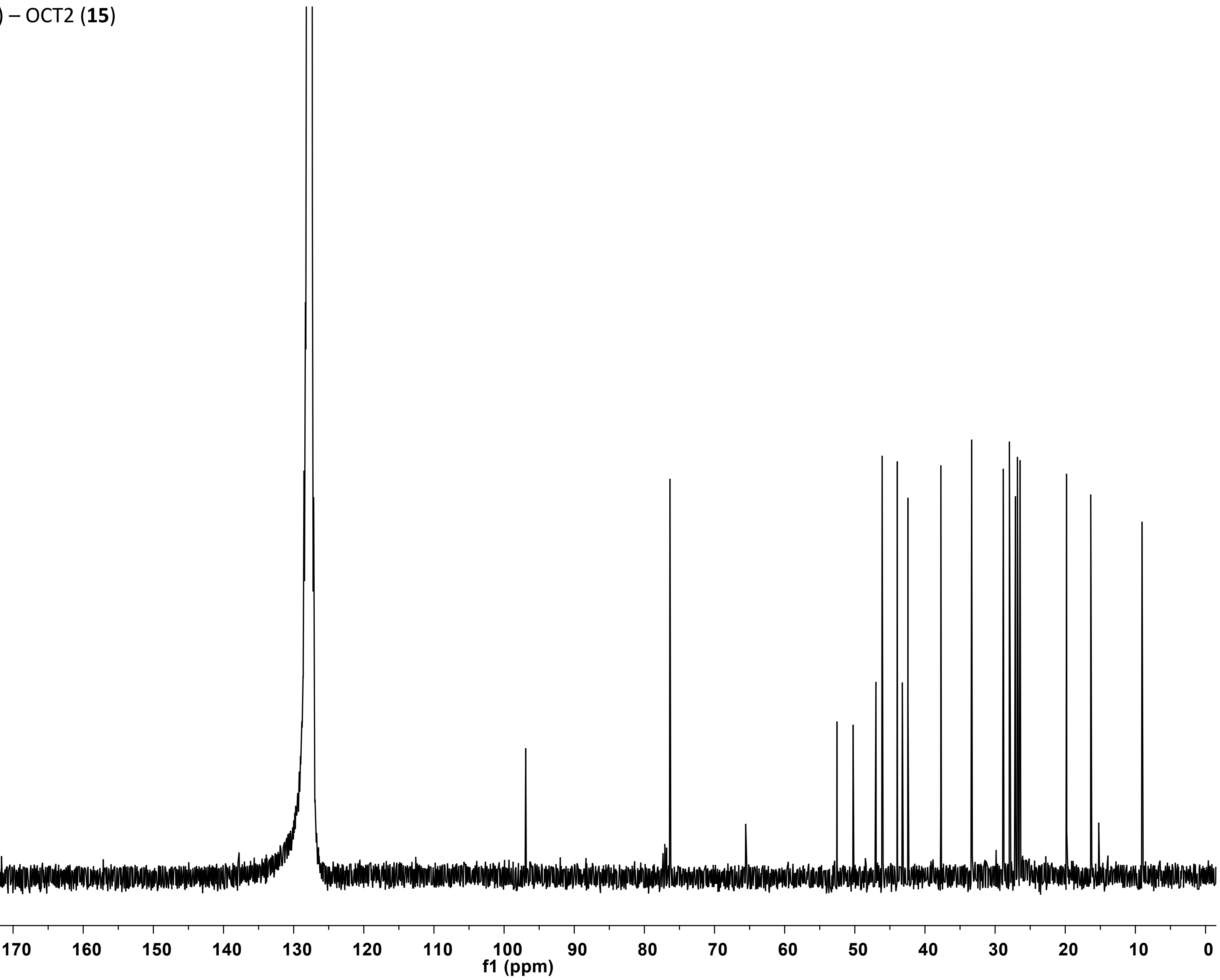

HMBC (500 MHz, C_6D_6)- OCT2 (**15**)

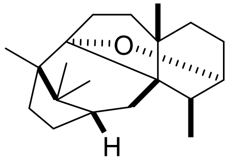

15

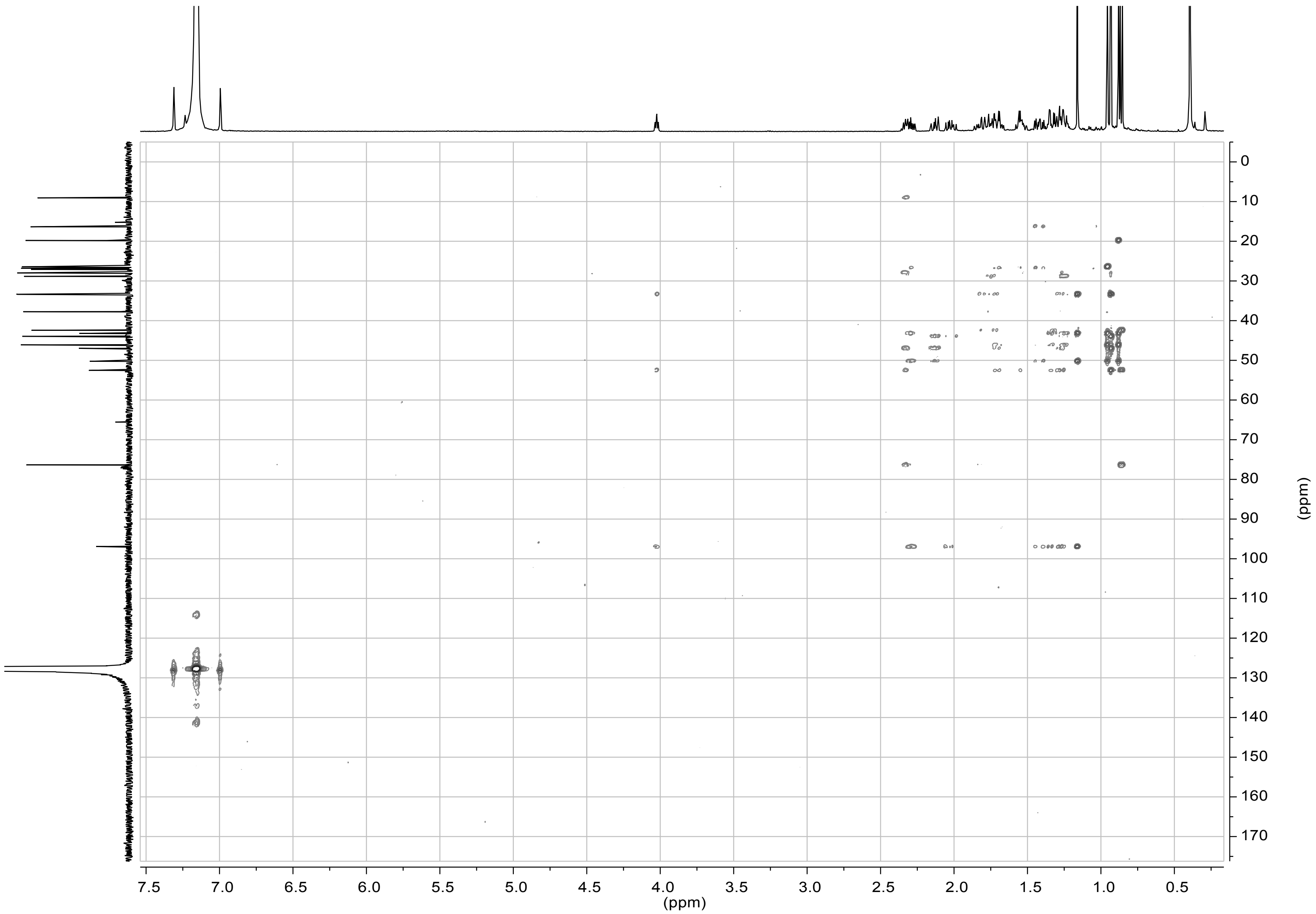

^{1}H COSY (500 MHz, C_6D_6)- OCT2 (**15**)

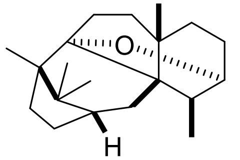

15

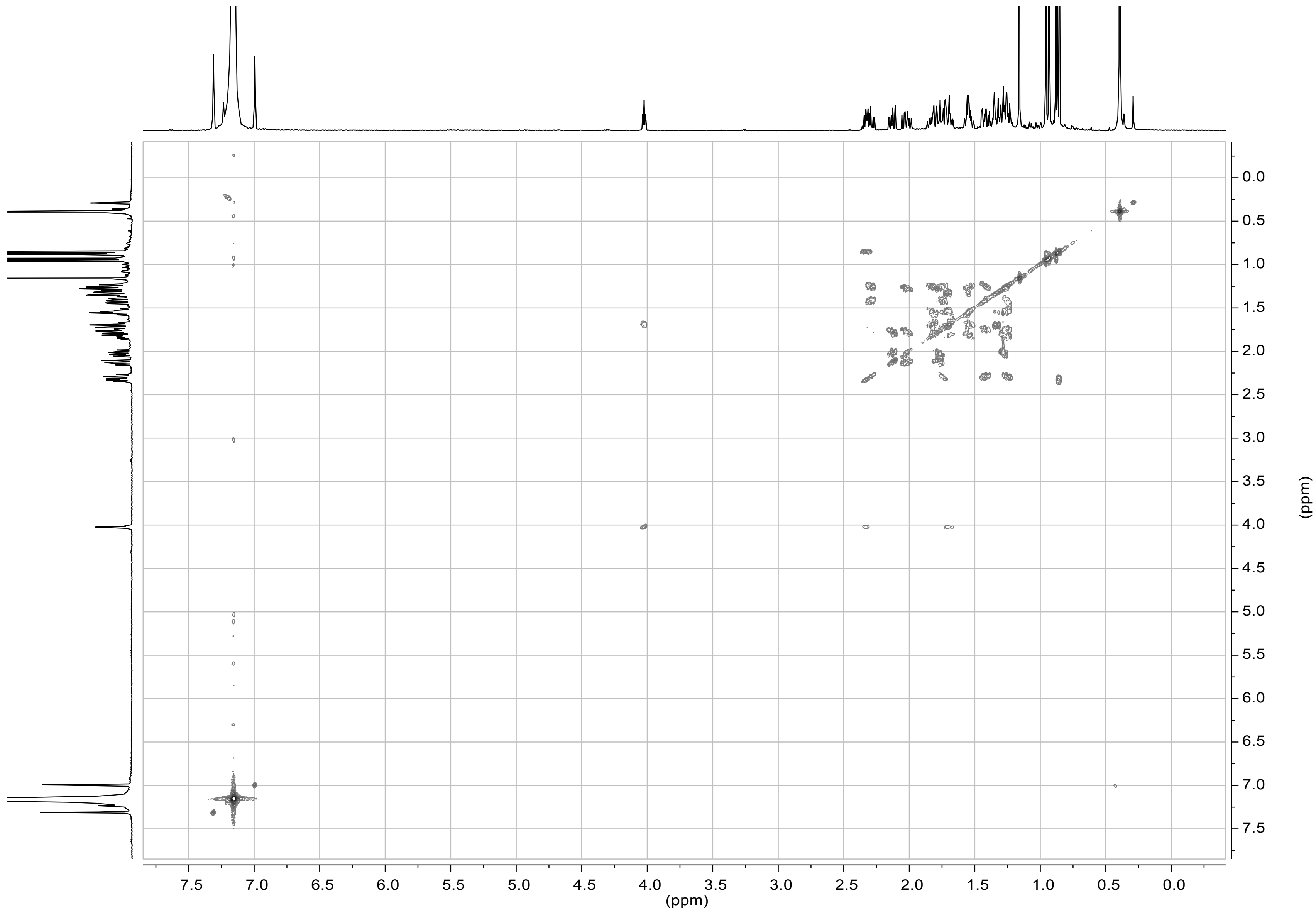

DEPT edited HSQC (500 MHz, C_6D_6)- OCT2 (**15**)

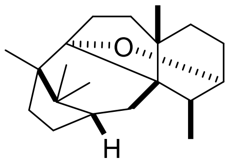

15

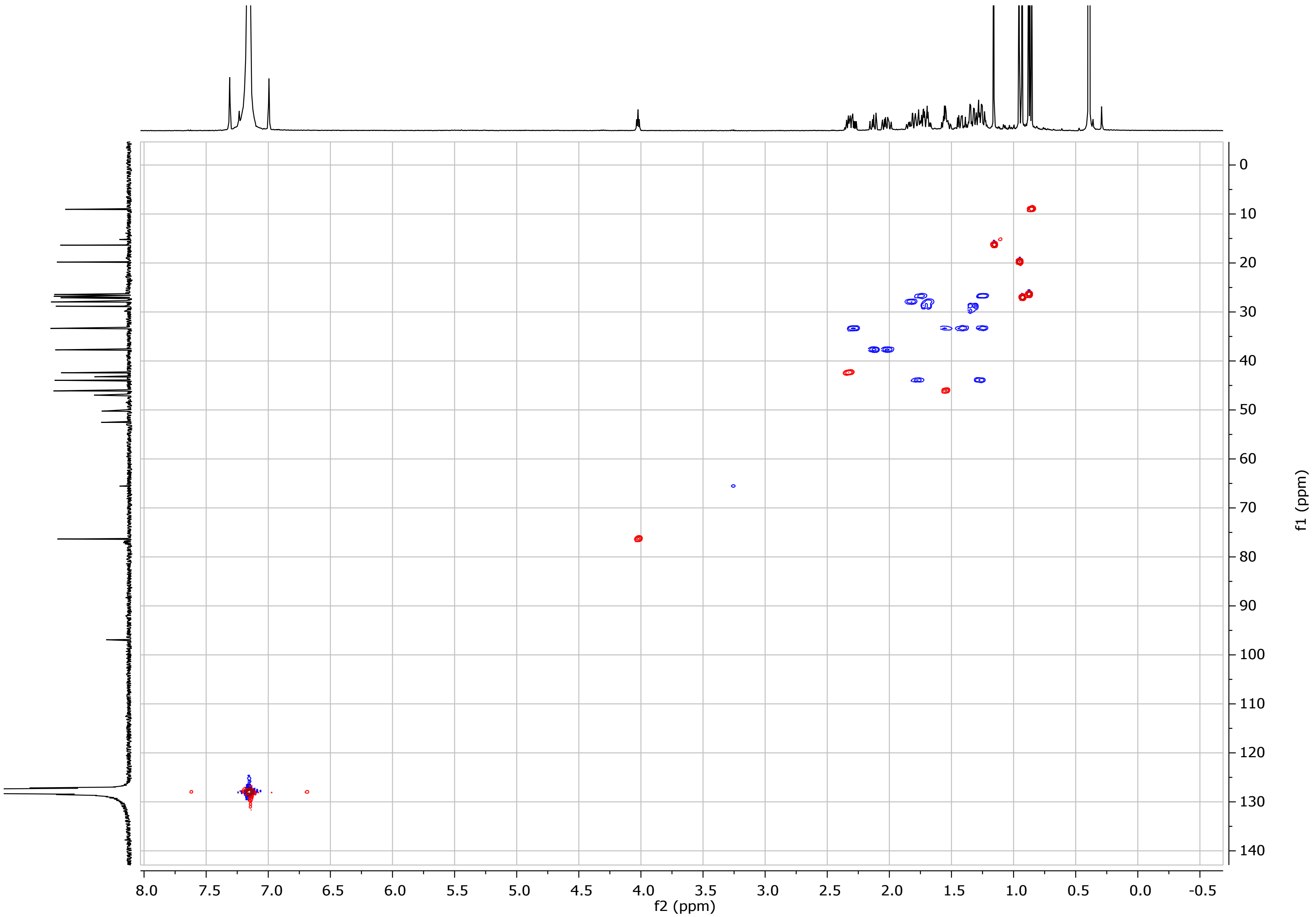

NOSEY (500 MHz, C_6D_6, 600 ms mixing time)- OCT2 (**15**)

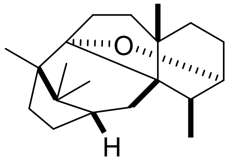

15

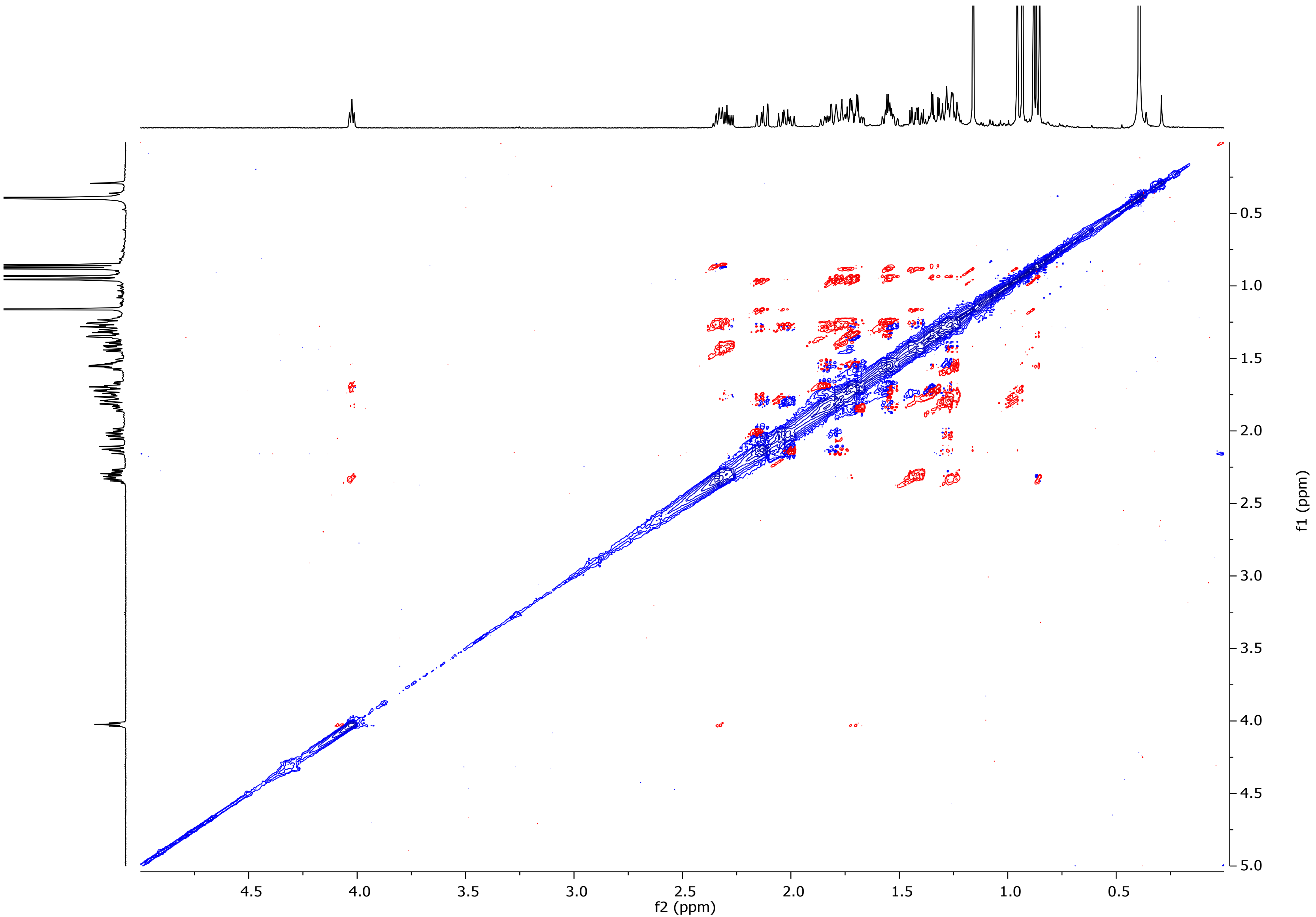

Table 2: ^{1}H and ^{13}C NMR assignments including COSY and HMBC correlations for OCT2 **15** (C_6D_6).

Position	^{1}H δ (ppm)[a]	COSY	HSQC	HMBC	^{13}C δ (ppm)
1	1.55	2a, 2b	46.1	2	46.1
2a	1.70	2b,	28.8	2	28.8
2b	1.33	2b,	28.8	15, 1, 3	28.8
3			52.5	2	52.5
4	2.33	20, 5	42.4	20, 6, 8, 3, 5	42.4
5	4.02	4, 6b	76.4	13, 3, 11	76.4
6a	1.82	6b, 7a	28.0	7	28.0
6b	1.69	5, 6a	28.0	8, 3	28.0
7a	1.55	6a, 7a	33.3	3	33.3
7b	1.25	7b	33.3		33.3
8			50.0	4, 10, 19	50.0
9a	1.77	10a	44.0		44.0
9b	1.27	10b	44.0	2, 8, 3, 11	44.0
10a	2.13	10b, 9a	37.7	9, 8, 12, 11	37.7
10b	2.02	10a, 9b	37.7	9, 11	37.7
11			96.9	5, 13, 10, 18	96.9
12			50.3	13, 10, 16, 17	50.3
13a	2.29	13b, 14a	33.3	14, 15, 12, 11	33.3
13b	1.42	13a, 14b	33.3	18, 14, 12, 1	33.3
14a	1.74	13a	26.8	2	26.8
14b	1.25	13b	26.8		26.8
15			43.2	16, 17, 18	43.2
16 or 17	0.95		19.8	16 or 17, 15, 1, 12	19.8
17 or 16	0.88		26.4	17 or 16, 15, 1, 12	26.4
18	1.16		16.4	13, 15, 12, 11	16.4
19	0.93		27.1	7, 9, 8, 3	27.1
20	0.86	4, 5	9.1	4, 3, 5	9.1

[a] 1H ppm determined from HSQC.

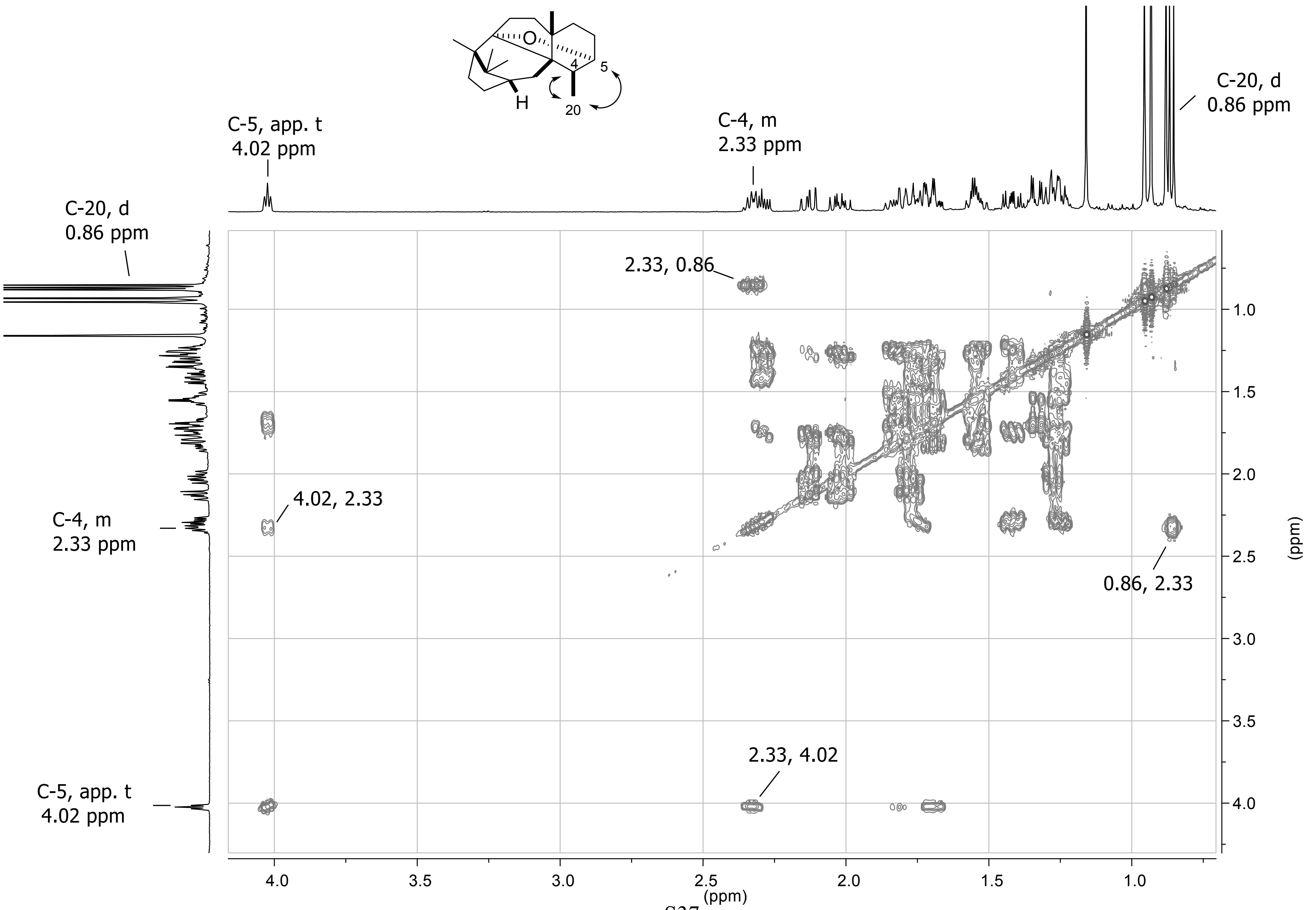

O
H
4
5
20
C-5, app. t
4.02 ppm
C-4, m
2.33 ppm
C-20, d
0.86 ppm
2.33, 0.86
4.02, 2.33
0.86, 2.33
2.33, 4.02
(ppm)

HMBC (500 MHz, C_6D_6) – OCT2 (**15**)

Showing key methyl correlations

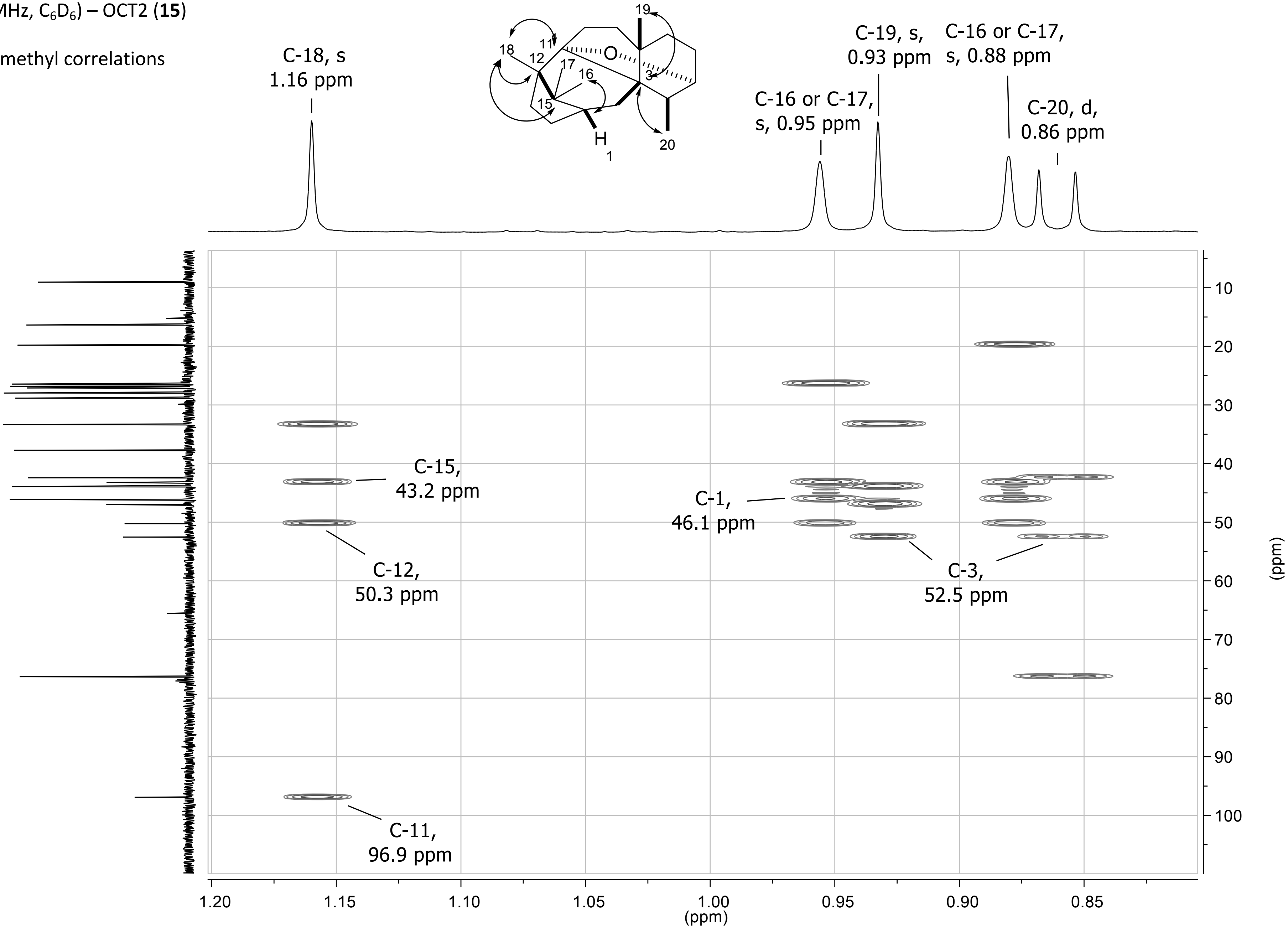

Before (**A**) and after (**B**) treatme

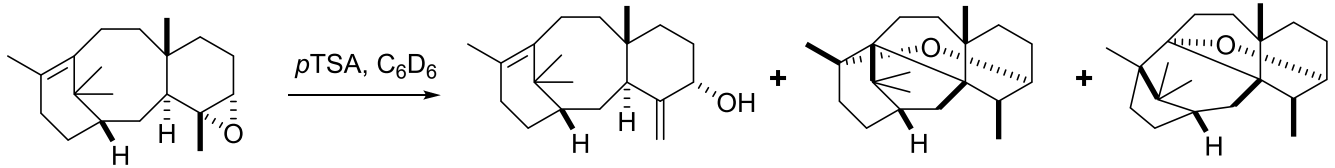

12 **4** **5** **15**

^{1}H NMRs (400 MHz, C_6D_6):

A: crude taxadiene-4(5)-epoxide (**12**) and remaining taxadiene (**3** and **6**)

B: crude reaction after treatment

with TsOH.

D: OCT2 (**15**)

C: OCT (**5**)

^{1}H NMRs (400 MHz, C_6D_6) – cyclogeraniol-derived epoxide (**23**):

Before (**A**) and after (**B**) treatment with Fe(TPP)Cl

D: Taxa-4(20), 11(12)-dien-5α-ol (**4**)

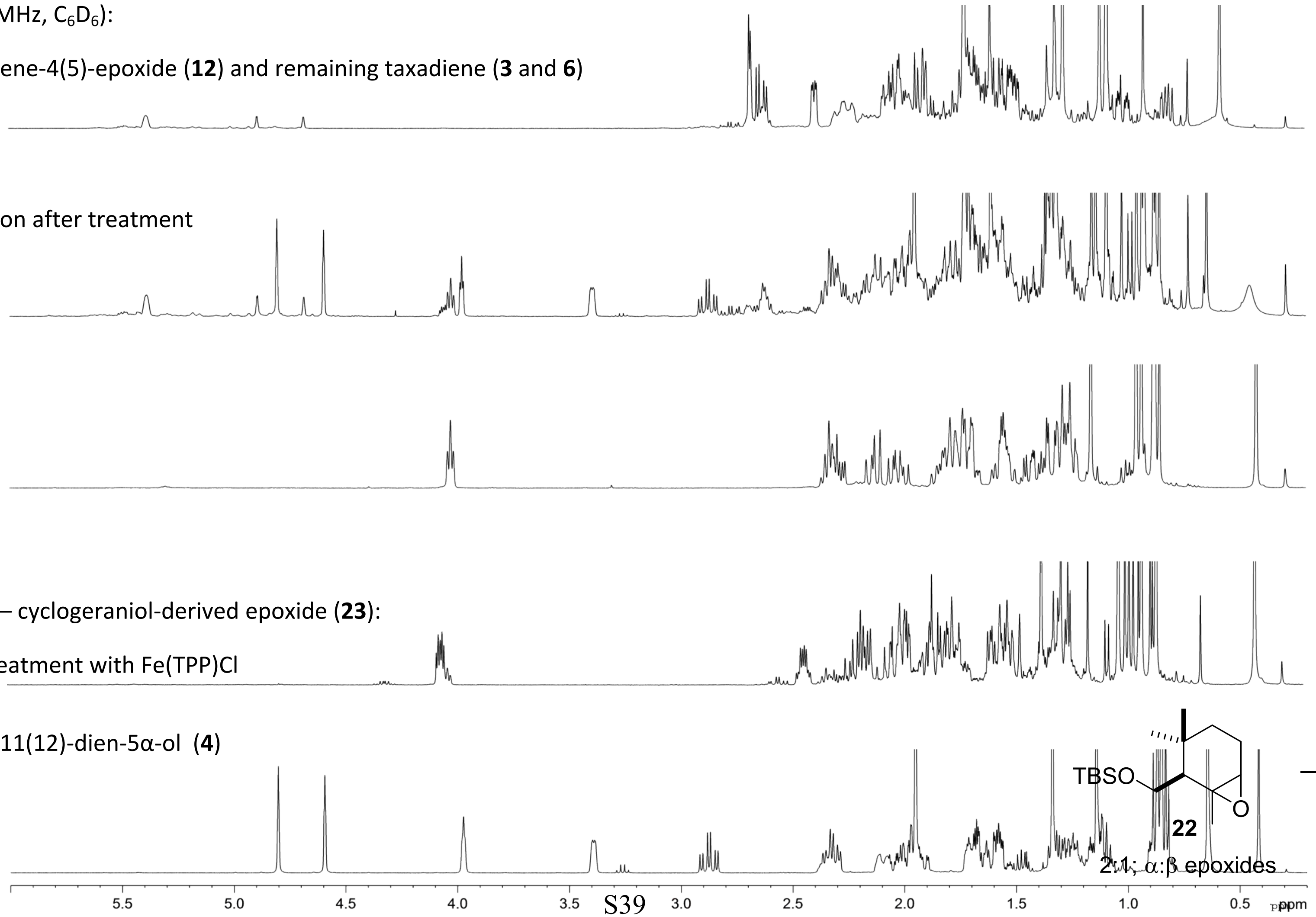

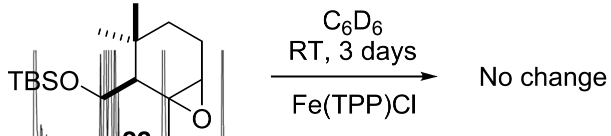

22

2:1; α:β epoxides

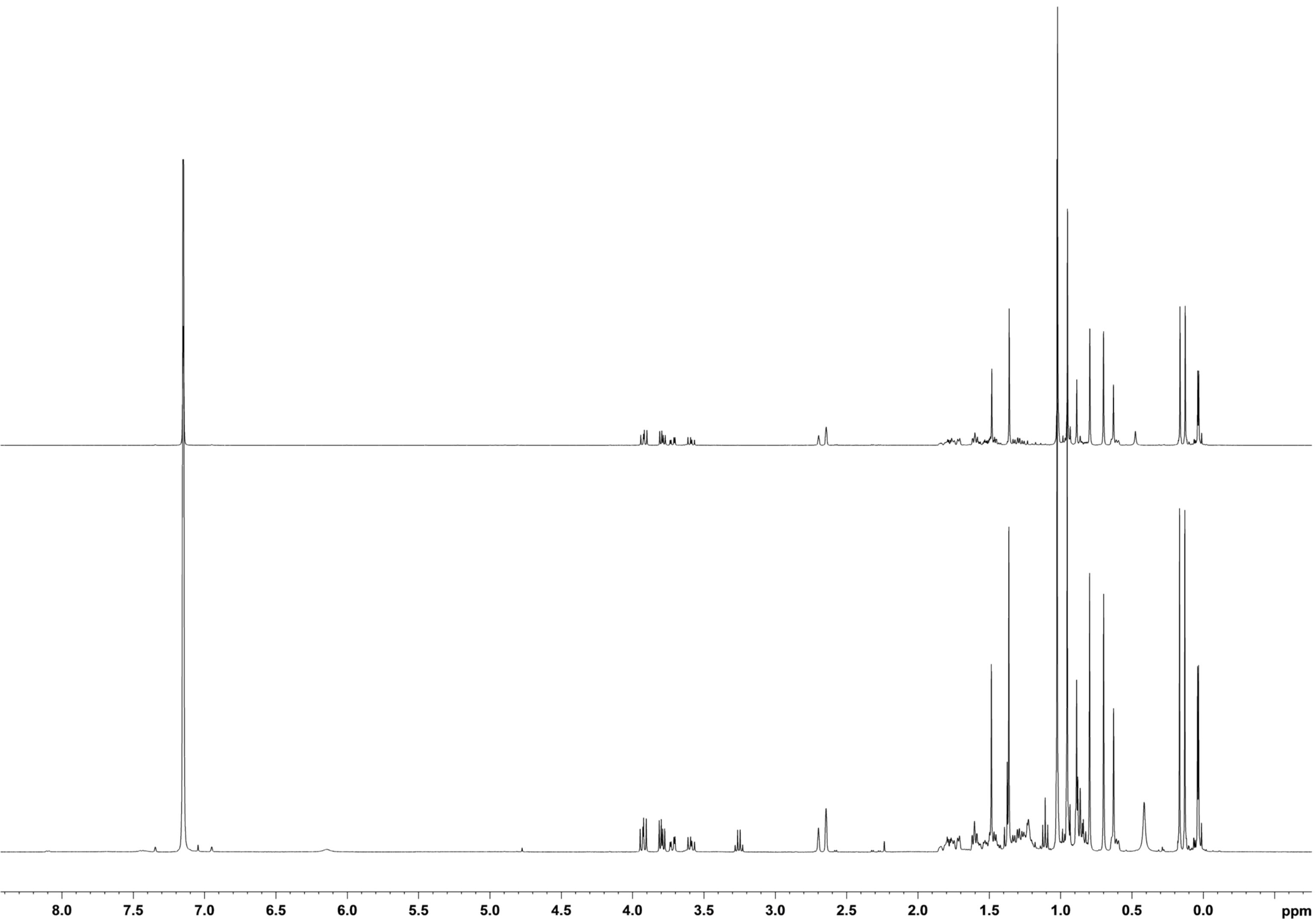

A: epoxide (**22**) before Fe(TPP)Cl treatment

B: epoxide (**22**) after Fe(TPP)Cl treatment

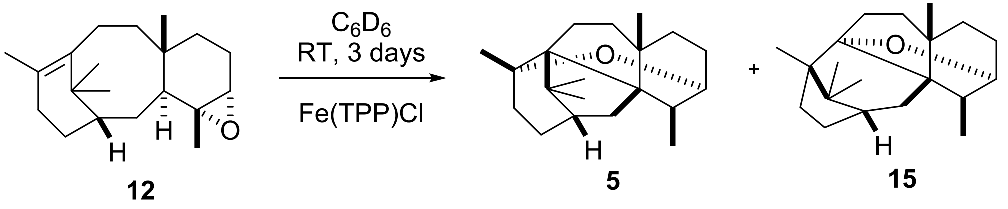

^{1}H NMRs (400 MHz, C_6D_6):

A: crude taxa-4(5)-epoxide (**12**) and remaining taxadiene (**3** and **6**)

B: crude reaction after treatment with Fe(TPP)Cl

C: OCT (**5**)

D: OCT2 (**15**)

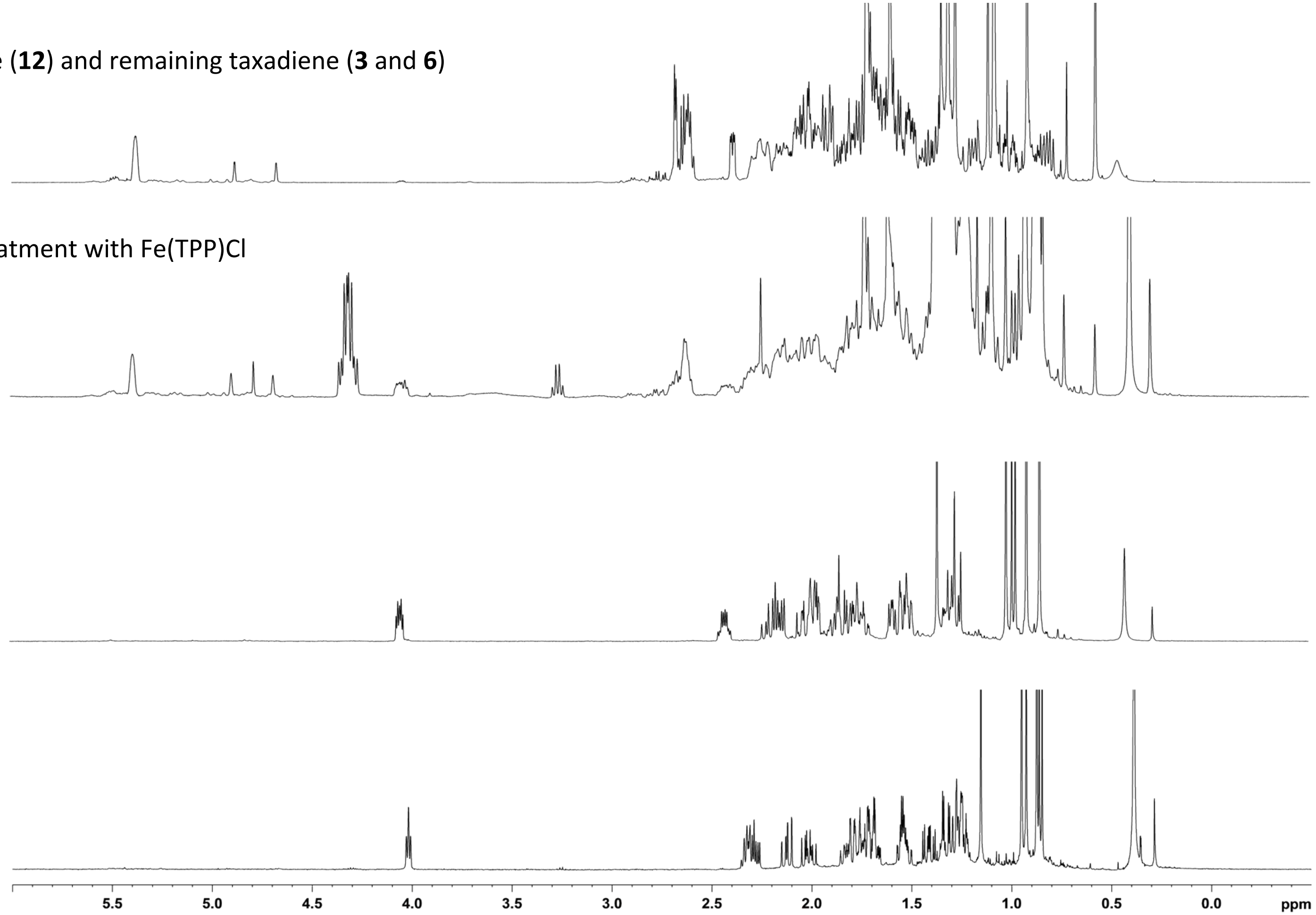

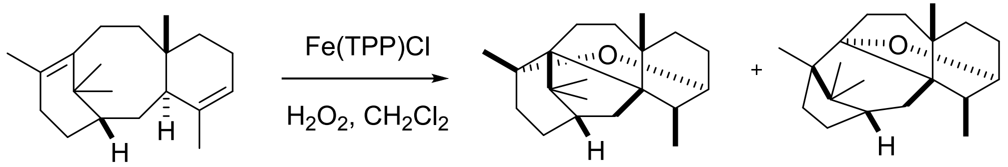

3 **5** **15**

^1H NMRs (400 MHz, C_6D_6):

A: Taxadiene (**3** and **6**)

B: crude reaction after treatment with Fe(TPP)Cl/ H_2O_2

C: OCT (**5**)

D: cyclic ether (**15**)

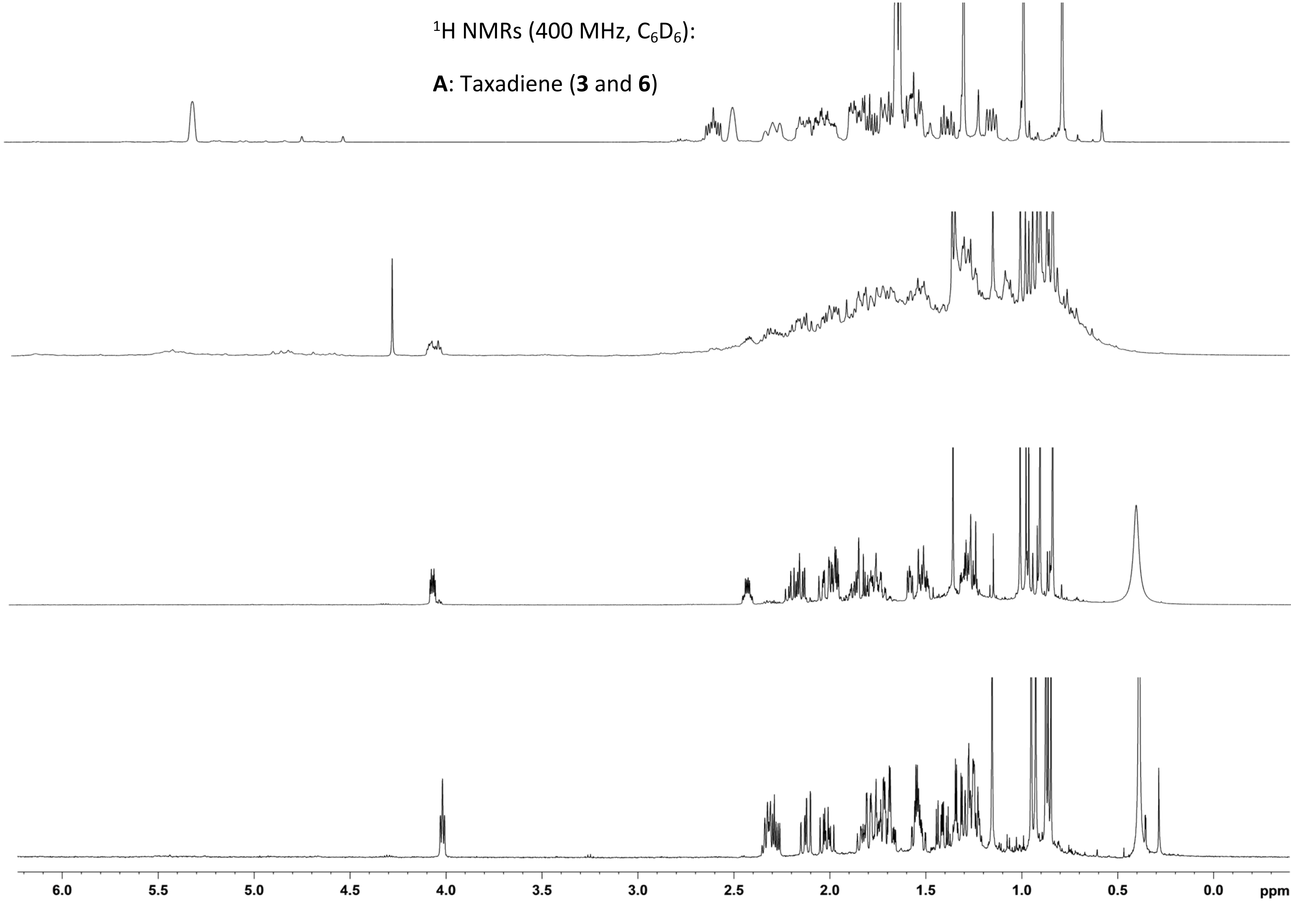

Supplement: Supplementary file 1 [file SC-007-C5SC03463A-s001.pdf]
